# *Histoplasma capsulatum* Heat-Shock 60 Orchestrates the Adaptation of the Fungus to Temperature Stress

**DOI:** 10.1371/journal.pone.0014660

**Published:** 2011-02-10

**Authors:** Allan Jefferson Guimarães, Ernesto S. Nakayasu, Tiago J. P. Sobreira, Radames J. B. Cordero, Leonardo Nimrichter, Igor C. Almeida, Joshua Daniel Nosanchuk

**Affiliations:** 1 Division of Infectious Diseases, Department of Medicine, Albert Einstein College of Medicine of Yeshiva University, Bronx, New York, United States of America; 2 Department of Microbiology and Immunology, Albert Einstein College of Medicine of Yeshiva University, Bronx, New York, United States of America; 3 Pacific Northwest National Laboratory, Richland, Washington, United States of America; 4 Group of Computational Biology, Laboratory of Genetics and Molecular Cardiology, Heart Institute (InCor), São Paulo, Brazil; 5 Laboratório de Estudos Integrados em Bioquímica Microbiana, Instituto de Microbiologia Professor Paulo de Góes, Universidade Federal do Rio de Janeiro, Rio de Janeiro, Brazil; 6 Department of Biological Sciences, The Border Biomedical Research Center, University of Texas at El Paso, El Paso, Texas, United States of America; Indiana University, United States of America

## Abstract

Heat shock proteins (Hsps) are among the most widely distributed and evolutionary conserved proteins. Hsps are essential regulators of diverse constitutive metabolic processes and are markedly upregulated during stress. A 62 kDa Hsp (Hsp60) of *Histoplasma capsulatum* (Hc) is an immunodominant antigen and the major surface ligand to CR3 receptors on macrophages. However little is known about the function of this protein within the fungus. We characterized Hc Hsp60-protein interactions under different temperature to gain insights of its additional functions oncell wall dynamism, heat stress and pathogenesis. We conducted co-immunoprecipitations with antibodies to Hc Hsp60 using cytoplasmic and cell wall extracts. Interacting proteins were identified by shotgun proteomics. For the cell wall, 84 common interactions were identified among the 3 growth conditions, including proteins involved in heat-shock response, sugar and amino acid/protein metabolism and cell signaling. Unique interactions were found at each temperature [30°C (81 proteins), 37°C (14) and 37/40°C (47)]. There were fewer unique interactions in cytoplasm [30°C (6), 37°C (25) and 37/40°C (39)] and four common interactions, including additional Hsps and other known virulence factors. These results show the complexity of Hsp60 function and provide insights into Hc biology, which may lead to new avenues for the management of histoplasmosis.

## Introduction

Heat shock proteins (Hsps) are among the most evolutionary highly conserved proteins across all species [1]. They are classified according to their relative molecular weight, comprising six major groups: small Hsps, Hsp40, Hsp60, Hsp70, Hsp90 and Hsp110. Hsps are ubiquitously expressed and often their levels are markedly upregulated as a key component of the heat shock (stress) response that occurs when a cell is exposed to challenging conditions (e.g. high temperature, oxidative stress, radiation, inflammation, exposure to toxins, starvation, hypoxia, nitrogen deficiency or water deprivation) [2]. Although the mechanisms by which heat shock (or other environmental stressors) activates the heat shock response has not been fully elucidated, some studies suggest that an increase in damaged or abnormal proteins activate Hsps [3].

Hsps have been termed molecular chaperones that are essential for maintaining cellular functions, including playing crucial roles in protein folding/unfolding, preventing aggregation of nascent polypeptides and toxicity by facilitating protein folding, directing assembly and disassembly of protein complexes, coordinating translocation/sorting of newly synthesized proteins into correct intracellular target compartments, degradation of aged/damaged proteins via the proteasome, regulating cell cycle and signaling, and also protecting cells against stress/apoptosis [4], [5].

*Histoplasma capsulatum* (Hc), a cosmopolitan dimorphic fungal pathogen, express Hsps that participate during pathogenesis [6]. For instance, Hsp60, enriched at Hc cell wall, is the ligand recognized by the integrin CR3 (CD11b/CD18), expressed on the surface of macrophage/monocytes [7], [8] through which Hc attaches to and is internalized by the phagocytes. Hsp60 from Hc is also an immunogenic molecule and protective antibodies were generated by our laboratory to control murine histoplasmosis [9], [10]. Thus, Hsp60 appears to be essential during the infective process. An Hsp70 was also identified in Hc [11], [12], [13]. Recombinant Hsp70 elicits a cutaneous delayed-type hypersensitive response in mice; however, the proteins did not confere protection to Hc infection. Potential roles for fungal Hsp on pathogenesis have been suggested in other models. Hsp90 down regulates the yeast-hyphal transition in *Candida albicans*, a crucial step during disease establishment [14]. The mechanism is regulated by Hsf1 and involves the Ras-PKA pathway, which is modulated by chaperonins [15]. This fact, in association with the presence of distinct Hsps in the cell wall strongly suggests that these proteins could be recruited to re-organize the fungal surface under stress conditions. Knock-down of innumerous proteins results in the loss of cell wall integrity [16], but there is no information concerning how these proteins interact to each other during cell wall reorganization such as involvement of Hsp.

Structural changes that occur during cell stress are potentially surpassed by Hsps remodeling [3], [4], [14], [17]. The importance of Hsp60 as a chaperone in fungi has been extensively studied in the model yeast *Sacharomyces cereviseae* [18], [19], [20], [21]. However, Hsps chaperone functions in a pathogenic fungus awaits characterization.

In the present work, we aimed to characterized Hc Hsp60-protein interactions under different temperatures to gain insights into the impact of Hsp60 on dimorphism, heat stress and pathogenesis, since it is known that the expression of Hc proteins is altered during conversion from mycelium to yeast phase and heat stress (40°C) [22]. We applied a proteomic approach to study the biological functions of Hsp60 by characterizing interactions of the protein with other fungal proteins. Our data suggests that specific Hc chaperone interactions are dependent on temperature and that they vary depending on subcellular location. We expect that dissection of the functional protein-protein Hsp60 interactions will lead to a better understanding of the cell biology of Hc and eventually provide a basis for novel therapeutic alternatives in the management of histoplasmosis.

## Materials and Methods

### Fungal strains and growth conditions

Hc strain G217B (obtained from the American Type Culture Collection, Rockville, Maryland, USA) [23] was grown in HAM F-12 medium (Invitrogen, Carlsbad, CA, USA) supplemented with 18.2 g/L glucose, 1.0 g/L D-glutamic acid, 84 mg/L cystine and 6.0 g/L HEPES at 30 and 37°C for 48 hours with 150 rpm in a rotatory shaker, to obtain the filamentous and yeast forms, respectively. Both phenotypes were monitored for morphology by light microscopy during growth. Yeast cells obtained from growth at 37°C were additionally incubated for 2 h at 40°C in a water bath to induce a heat-shock temperature stress.

### Isolation of cell extracts

Fractionation of proteins from yeast cells or hyphae obtained after growth under different conditions was performed as described [24], [25], with minor modifications. Cells were harvested by centrifugation at 1100×g for 10 min and washed three times with PBS (8 g/L of NaCl, 0.2 g/L of KCl, 0.2 g/L of NaH_2_PO_4_ and 1.2 g/L Na_2_HPO_4_, pH 7.2) containing protease inhibitor cocktail (Complete Protease Inhibitor Cocktail Tablets, Roche, Indianapolis, IN, USA). Subsequently, cells were suspended in ice-cold PBS and lysed by mechanical disruption using 0.5 mm zyrconia/silica beads (BioSpec Products, Bartlesville, OK, USA) in a bead-beater (BioSpec Products, Bartlesville, OK, USA). This procedure was carried out at 4°C until complete cell breakage was achieved, as verified by phase contrast microscopy and subsequently confirmed by absence of cell growth after plating of extracts on BHI blood agar [26]. Lysed cells were separated by centrifugation at 1100×g for 10 min into insoluble (cell wall) and soluble (cytoplasmic) fractions. Cell wall fractions were washed five times in cold PBS to eliminate intracellular and non-covalent linked proteins.

The cell wall crude extract was further digested under non-denaturing conditions using Novozyme (Calbiochem) and chitinase (Sigma-Aldrich, St. Louis, MO, USA), overnight at 37°C, under agitation, followed by centrifugation at 10000×g to eliminate insoluble material. For normalization, the volume of the cell wall extract was equalized to the volume of the cytoplasm extract. Protein concentration in the extracts was determined by a dye binding protein assay (Bio-Rad, NY, USA) with respect to a bovine albumin-globulin standard [27].

### Immunoblotting analysis of Hc extracts

Protein extracts were normalized by XTT activity in the cytoplasmic extracts. Electrophoresis and immunoblotting were conducted as previously described [28], [29]. Membranes were blocked using 5% skim milk solution in TBS. After 3 washes with TBS, extracts containing membranes were probed by incubating with 5 µg/mL of mAb 4E12 against Hsp60. A horseradish peroxidase-conjugated anti-mouse Ig (SouthernBiotech, Birmingham, AL, USA) diluted in blocking buffer was added and strips incubated for 1 h at 37°C. Membranes were developed using a SuperSignal West Pico Chemiluminescent Substrate (Thermo Fisher Scientific (Rockford, IL, USA). Equivalent loading of extracts was confirmed using an antibody against tubulin (Abcam, Cambridge, MA).

### Hsp60 capture ELISA

Hsp60 levels were measured by capture ELISA as previously described [30]. Briefly, polystyrene 96-well plates were coated with 50 µL of a 20 µg/mL IgG2a mAb 4E12 against the Hc Hsp60 [9] diluted in PBS for 1 h at 37°C. Plates were blocked with 200 µL 2% bovine serum albumin solution in TBS-T (50 mM Tris base, 150 mM NaCl, PH 7.4, 0.01% Tween-20). After washes, 50 µL of different concentrations of recombinant Hsp60 (rHsp60) ranging from 20 µg/mL to 0.16 µg/mL in a 1∶2 serial dilution in blocking buffer were added to each well. rHsp60 was prepared as described [31]. Concomitantly, 50 µL of serial dilutions of Hc extracts were added and the plates were incubated for 1 h at 37°C. Plates were washed and then incubated with 50 µL of a 20 µg/mL solution of an IgG1 anti-Hsp60 (11D1) for 1 h at 37°C. After three additional washes, 50 µL of a 1∶1000 dilution of goat anti-mouse Ig alkaline-phosphatase conjugated (SouthernBiotech, Birmingham, AL, USA) were added to each well and the plates were incubated for 1 h at 37°C. The reaction was developed after washing and subsequent addition of 50 µL of a 1 mg/mL of p-nitrophenyl phosphate. Plates were read at 405 nm and concentrations of Hsp60 in the extracts were calculated based on the rHsp60 standard curve.

### Co-immunoprecipitation

Agarose beads were cross-linked with Hsp60-binding mAb 4E12 using the Pierce Direct IP Kit according to manufacturer's protocol (Pierce, Rockford, IL, USA). Co-immunoprecipitation was performed by incubating 100 µg Hsp60 from each extract with 100 µL mAb 4E12-coupled resin, overnight at 4°C. This allowed us to determine the levels of interactions of the Hsp60 with its partners depending on the temperature. Controls were performed using beads coated with an irrelevant matched mAb (SouthernBiotech, Birmingham, AL, USA). Samples were centrifuged at 2500×g and the beads were washed five times with PBS. Protein complexes were eluted after addition of elution buffer (0.1 M glycine, pH 2.8) and centrifugation at 2500×g. To reduce non-specific interactions, this procedure was repeated a second time using the eluted proteins.

### Identification of proteins by immunoblotting

The captured and eluted proteins were subjected to SDS-PAGE, transferred to immunoblotting membranes, and probed with 5 µg/mL of monoclonal antibodies (mAbs) against the Hsp60 [9], H2B [26], Hsp70 [11] and M antigen (catalase 2B) [29] as described elsewhere [9].

### Identification of proteins by LC/MS-MS

Sample preparation for mass spectrometry was performed essentially as described elsewhere [32]. Briefly, lyophilized protein pull-downs were diluted with 200 µL of HPLC-grade water (Sigma-Aldrich, St. Louis, MO, USA) and trichloroacetic acid was added to a 10% final concentration. Samples were incubated for 30 min at room temperature and centrifuged at 16000×g for 20 min at 4°C. Pellets were washed with ice-cold acetone, centrifuged at 16000×g for 5 min and dried in a chemical hood. Dried material was suspended in 20 µL 0.4 M NH_4_HCO_3_ containing 8 M urea. Disulfide bonds were reduced by the addition of 5 µL 45 mM dithiotreitol at 50°C for 15 min, and the samples were alkylated by the addition of 5 µL iodoacetamide for 15 min at room temperature protected from the light. Samples were diluted to a final urea concentration of 1 M using HPLC-grade water and digested with 5 µg proteomic-grade trypsin (Sigma-Aldrich, St. Louis, MO, USA), at 37°C for 24 h. Samples were desalted in a C-18 reverse phase zip-tip (POROS R2, Applied Biosystems) [33]. Zip-tips were washed twice with 100 µL 0.046% of trifluoroacetic acid (TFA), and peptides were eluted with 100 µL 80% acetonitrile containing 0.046% TFA, dried in a vacuum centrifuge (Eppendorf, Hauppauge, NY, USA) and suspended in 30 µL 5% acetonitrile (ACN)/0.5% formic acid (FA). Peptides (10 µL) were loaded onto an in-house C18 trap column (2 cm×75 µm, packed with Luna 5-µm C18 resin, Phenomenex) and washed for 10 min with the 5% ACN/0.5% FA buffer. The separation was performed in a capillary reverse phase column (20 cm×75 um, in-house packed with Luna 5-µm C18 resin, Phenomenex) connected to a nanoHPLC system (nanoLC 1D plus Eksigent). Elution of peptides was performed in a 5–40% gradient of solvent B (solvent A: 5% ACN/0.1% FA; solvent B: 80% ACN/0.1% FA) during 200 min at a flow rate of 200 nL/min. Eluting peptides were directly analyzed in an ESI-linear ion-trap mass spectrometer (LTQ XL with ETD, Thermo Fisher Scientific, San Jose, CA). Samples were injected into the LTQ XL using an automated nanoinjection system (Triversa Nanomate, Advion, Ithaca, NY) set at 1.35 kV. MS spectra were collected in positive-ion mode at the 400–1700 mass-to-ratio (*m/z*) range. The 10 most abundant ions were submitted to CID (35% normalized colision energy). Peptides within the 800–3500 Da range were fragmented (MS/MS). Spectra were converted to DTA files using Bioworks v.3.3.1 (Thermo Fischer Scientifics, Waltham, MA) and searched against a Hc database (version 10/24/08, http://www.broadinstitute.org/annotation/genome/histoplasma_capsulatum/download/?sp=EAProteinsFasta&sp=SHC1&sp=S.zip; including 9251 Hc proteins) using TurboSequest [34], Bioworks software. Common contaminant sequences, such as human keratin, bovine trypsin and mouse IgG2a, retrieved from GenBank (http://www.ncbi.nlm.nih.gov/), were also included in the database. The database was concatenated with its reverse version totalizing 20,512 sequences. Database search parameters were the following: trypsin as the digesting enzyme (one missed cleavage site allowed); carbamidomethylation of cysteine residues and oxidation of methionine residues as fixed and variable modifications, respectively; 2.0 Da and 1.0 Da for peptide and fragment tolerance, respectively. To ensure the quality of the identifications, Bioworks was set with the following filters: DCn <0.085, protein probability <1e−3, Xcorr ≥1.5, 2.2 and 2.7 for peptides with single, double and triple positive charges, respectively. Considering these parameters, the calculated FRP value was 0.93%.

### Results interpretation and database organization

Identified proteins in each extract from the different temperature conditions were grouped and classified according to their biological function as described [35]. Proteins were automatically annotated using the Blast2go software (www.blast2go.org/) and manually checked according to the UCSF HistoBase (http://histo.ucsf.edu/) and UniProt Protein Database (http://www.uniprot.org/). Briefly, proteins were divided into groups: amino acid metabolism; protein metabolism and modification; carbohydrate metabolism; lipid, fatty acid and steroid metabolism metabolism; nucleoside, nucleotide and nucleic acid metabolism; cell growth/division; nuclear; cell signaling; cytoskeletal; cell wall architecture; plasma membrane; anti-oxidant; proteasome component; chaperone-like; ribosomal; miscellaneous or unclassified biological process. The analyzed proteins were grouped by temperature and cell localization using the Osprey Network Visualization System (Mount Sinai Hospital, Toronto, Canada).

Subsequently, hits identified by mass spectrometry were further filtered and the data were checked for enriched proteins forming a complex with Hsp60 [36], We also assessed for enrichment in published interactors using the BioGRID database [37]. We considered only LC-MS/MS data with confidence scores higher than 90% [38].

### Quantitative MS analysis

Quantitative MS was performed as previously described [39], [40]. Briefly, spectral counts were retrieved from MS data, enumerated, and correlated with relative abundance of each identified protein. Additional analyses were performed with *e*xponentially *m*odified *p*rotein *a*bundance *i*ndex (emPAI), which is a value obtained from PAI (number of observed peptides divided by the number of observable peptides). Quantitative parameters were correlated by Pearson correlation using Prism 5 for WindowsVersion 5.02 (GraphPad Software, Inc.). emPAI values were used to determine the percentage of association of proteins with the Hsp60 under all the conditions.

## Results

### Temperature stress induces upregulation of Hsp60 in cell wall and cytoplasmic fractions of *H. capsulatum*

**Figure 1:**
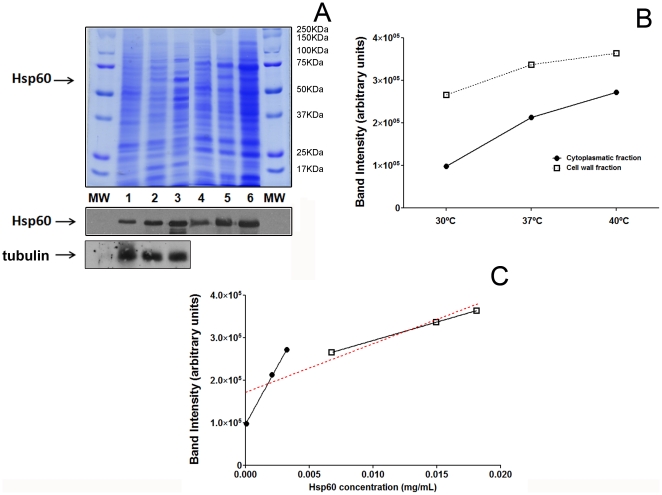
Analysis of Hsp60 levels in cellular fractions under distinct temperature conditions. (A) SDS-PAGE displays the composition of proteins in the cytoplasm and cell wall of Hc under the different temperature conditions evaluated (upper panel). Notably, the levels of Hsp60 increase with temperature stress (immunoblot, lower panel). Lanes 1,2 and 3 are from cytoplasmic extracts obtained from cultures grown at 30, 37 and 37/40°C, respectively; 4, 5 and 6- cell wall preparations of yeast grown at 30, 37 and 37/40°C, respectively. MW represents the molecular marker. The samples were normalized as described in the methods. (B) Densitometry shows that Hsp60 levels increase with temperature stress in both cytoplasm and cell wall preparations. (C) Correlations of measured Hsp60 levels obtained by ELISA (Table 1) and immunoblot (p<0.05).

The levels of Hsp60 in the cellular fractions obtained under different growth conditions were measured by capture ELISA. Levels of Hsp60 were higher in extracts from the cell walls than from cytoplasm under all temperature conditions. However, for any given subcellular fraction evaluated, Hsp60 levels increased with temperature stress (Table 1). Densitometric analysis of immunoblot bands showed similar increases in Hsp60 with increasing temperatures (Figure 1A and B). Doublet or triplets of Hsp60 ranging from 64–53 kDa are frequently obtained in cell wall extracts, as previously described [41]. A representative correlation is described in Figure 1C, displaying a concordance between Hsp60 detection by ELISA and immunoblot (p =  0.021, Pearson r = 0.77).

**Table 1:** Concentration of Hsp60 in cytoplasm and cell wall under different temperature conditions.

Cellular Fraction	Temperature(^o^C)	ELISA(µg/mL)	Immunoblot band intensity(arbitrary units)
Cytoplasm	*30*	0.050	31.8
	*37*	2.1	69.2
	*37/40*	3.3	88.1
Cell Wall	*30*	6.7	86.1
	*37*	14.9	108.8
	*37/40*	18.1	118.0

### Hc Hsp60 interacts with Hsp70, M antigen and H2B

**Figure 2:**
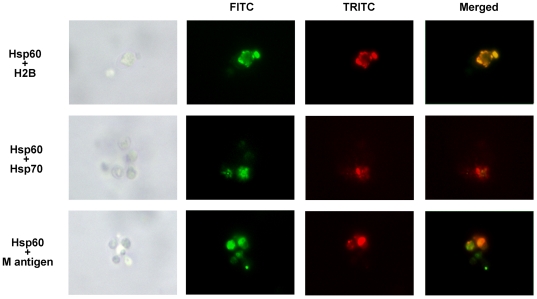
Immunofluorescence images depicting the co-localization of Hsp60 with either H2B, M antigen or Hsp70 on *H. capsulatum* yeast cells. FITC indicates the presence of mAb to Hsp60, whereas TRITC represents mAb to H2B, M antigen or Hsp70.

Histone 2B (H2B), Hsp70 and M antigen are described cell wall antigens of Hc and are protein involved in pathogenesis of histoplasmosis as previously described by our group [11], [13], [26], [29]. We investigated whether H2B, Hsp70 and M antigen co-localize with Hsp60 by immunofluorescence, which would suggest potential interactions. A diffuse pattern of co-localization was observed at 37°C with H2B, Hsp70 and M antigen (Figure 2).

Hsp60 association with these proteins was prominent at the cell wall level, and protein complexes were observed in clusters. The punctate co-localization pattern along the cell wall was consistent with the presence of these proteins within vesicular structures, as previously described [35].

### Co-immunoprecipitation with Hsp60 mAbs reveals differential interactions associated with temperature stress

**Figure 3:**
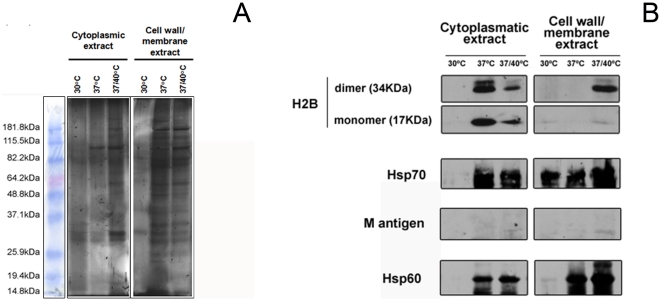
Co-immunoprecipitation identifies proteins partners that display distinct patterns in the different cellular fractions and temperature conditions. (A) Representative SDS-PAGE gel of pull down samples obtained after co-immunoprecipitation of extracts. The experiment was repeated three times with consistent results. (B) Immunoblots with mAbs against the H2B, M antigen and Hsp70 indicated the presence of these proteins in the extracts.

Protein extracts eluted from agarose beads coated with Hsp60-binding mAb were subjected to SDS-PAGE and silver-stained. In all extracts, several protein bands were observed, ranging from 250 to 10 kDa (Figure 3A). Hsp60 interacted with more proteins in the cell wall extracts than in cytoplasm extracts. However, a distinct pattern of interactions was observed for each temperature when the same cellular compartment was analyzed. Although the number of interacting proteins increased significantly with temperature rise, common bands were observed in the cytoplasm at 37 and 37/40°C. The finding that there were more Hsp60-interacting partners with the cell wall extracts with increasing temperature suggests that the trafficking activity of this protein and localization of several proteins to this organelle is augmented at high temperature stress condition.

Pull-downs of proteins were also subjected to SDS-PAGE, transferred to nitrocellulose membranes and immunoblotted against the antibodies to the described surface proteins H2B, Hsp70 and M antigen. H2B and Hsp70 were found to interact with Hsp60 in the cytoplasm and cell wall of yeast in each condition evaluated (Figure 3B). M antigen was found in the cytoplasm and cell wall 37°C and 37/40°C pull-downs, but it was observed only in cell walls extracts at 30°C.

### Identification of the Hsp60 interactome by tandem mass spectrometry shows common and specific interactions of Hc Hsp60 that vary with temperature and subcellular location

**Figure 4:**
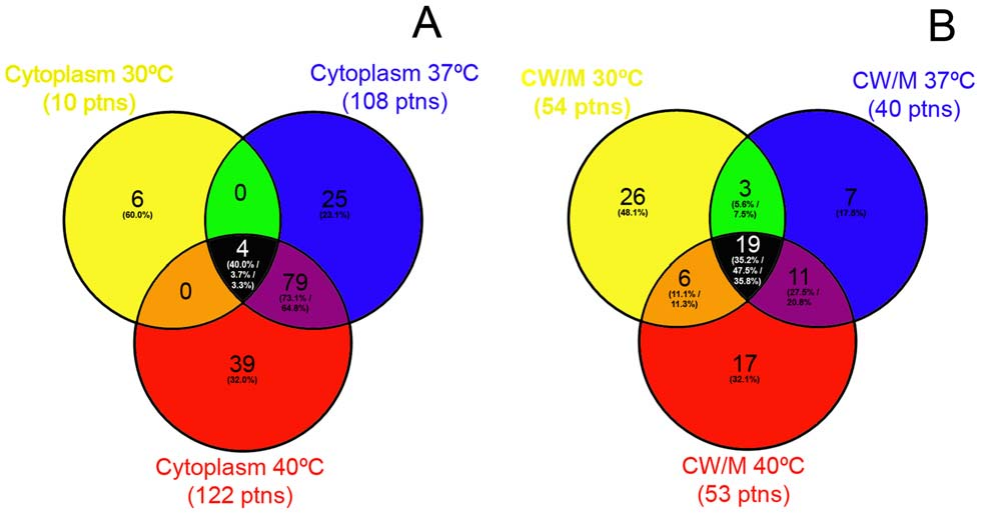
Common (intersections) and specific interactions of Hc Hsp60 according to temperature and location in (A) cytoplasm or (B) cell wall fractions.

Total proteins interacting with the Hsp60 were identified in both cytoplasmic and cell wall subcellular fractions. In the cytoplasm, Hsp60 interacted with 10 proteins at 30°C, 108 proteins at 37°C and 122 proteins at 37/40°C (Figure 4A). Analysis of cytoplasmic fractions showed the presence of 4 common interaction partners of Hsp60 (including H2B and Hsp70) within the cytoplasm at the different temperature conditions, comprising 40, 3.7, and 3.3% of all the interactions observed at 30, 37, and 37/40°C, respectively. Specific interactions were also identified within each temperature conditions in this subcellular fraction, accounting for 6 specific interactions at 30°C (60%), 25 interactions at 37°C (23.1%), and 39 interactions at 37/40°C (32%).

For the cell wall fractions, a significantly higher number of interaction partners were observed. Hsp60 interacted with 54 proteins at 30°C, 40 proteins at 37°C and 53 proteins at 37/40°C (Figure 4B). Analysis revealed 19 common partners were identified in the temperature conditions evaluated (including H2B, Hsp70 and M antigen), comprising 35.2, 47.5, and 35.8% at 30, 37, and 37/40°C, respectively. Specific interactions in this cellular fraction were also observed, accounting for 26 partners of interactions at 30°C (48.1%), 7 interactions at 37°C (17.5%), and 17 interactions at 37/40°C (32.1%).

### Hsp60 participates in the Hc stress response

**Figure 5:**
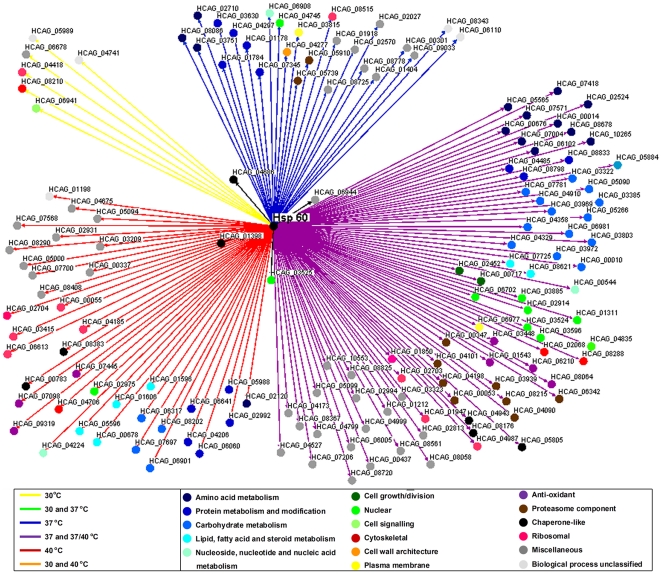
Map of Hsp60 interaction with cytoplasmic proteins at different temperatures. Colored dots display the metabolic classifications of the interacting proteins and colored lines represent the different temperatures. The interacting proteins are described in table S1.

The list of identified proteins was electronically annotated according to the UCSF HistoBase (http://histo.ucsf.edu/) and UniProt Protein Database (http://www.uniprot.org/). All proteins indentified under different temperature condition from both subcellular fractions analyzed were classified according to their metabolic function (Table 2). We used the Osprey Network Visualization System to group the Hsp60 interaction proteins according to their metabolic functions and temperature conditions, in order to construct a temperature-dependent interactome of Hc Hsp60. In cytoplasmic fractions, the number of Hsp60 interactions increased significantly with temperature stress, specifically for proteins involved in amino acid, protein, carbohydrate, lipid and nucleotide metabolism, nuclear proteins, anti-oxidant proteins, proteasome components, chaperones and ribosomal proteins (Figure 5, Table 2).

**Table 2:** Distribution of the Hsp60 interacting proteins in cytoplasm and cell wall according to their biological functions.

Biological Function	Cytoplasmic fraction			Cell wall fraction		
	30°C	37°C	40°C	30°C	37°C	40°C
Amino acid metabolism	X*	**13**	**11**	*3*	*1*	*1*
Protein metabolism and modification	**X**	**8**	**8**	**3**	**6**	**8**
Carbohydrate metabolism	**X**	**14**	**18**	**7**	**9**	**9**
Lipid, fatty acid and steroid metabolism	**X**	**2**	**6**	*3*	*X*	*X*
Nucleoside, nucleotide and nucleic acid metabolism	**X**	**2**	**2**	X	X	X
Cell growth/division	X	2	2	1	1	X
Nuclear	**1**	**9**	**9**	2	1	3
Cell signaling	1	X	X	X	X	X
Cytoskeletal	1	2	3	3	1	X
Cell wall architecture	X	1	X	3	1	1
Plasma membrane	X	2	1	1	1	2
Anti-oxidant	**X**	**4**	**7**	*2*	*X*	*X*
Proteasome component	**X**	**10**	**8**	**X**	**X**	**1**
Chaperone-like	**2**	**5**	**7**	**4**	**5**	**7**
Ribosomal	**1**	**5**	**9**	14	6	10
Miscellaneous	2	27	30	8	8	10
Uncharacterized protein	2	2	1	X	X	1
**Total**	10	108	122	54	40	53

Bold type represents a significant (p<0.05) increase in the specific protein category with increasing temperature.

Italics represent a significant (p<0.05) decrease in the specific protein category with increasing temperature.

X* indicates that no proteins within this category were detected.

**Figure 6:**
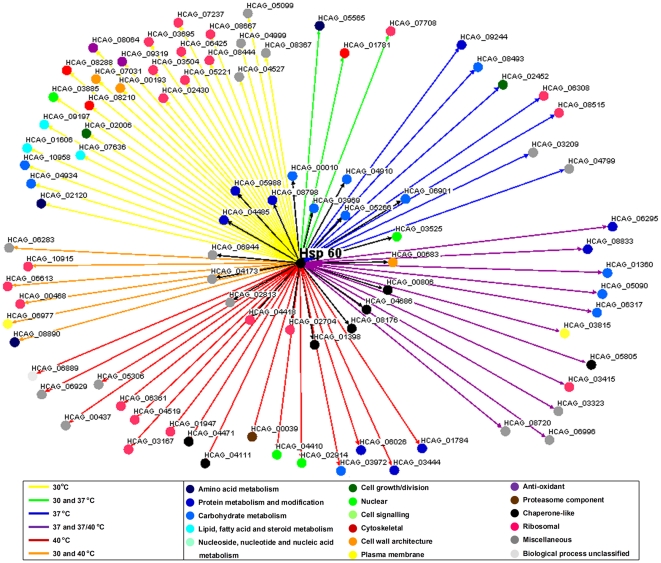
Map of Hsp60 interaction within cell wall at different temperatures. Colored dots display the metabolic classifications of the interacting proteins and colored lines represent the different temperatures. The interacting proteins are described in table S2.

A different number of Hsp60 interactions was observed in the cell wall compared to cytoplasm extracts at each temperature evaluated, although there was, in general, less variation in interactions at different temperatures (Figure 6, Table 2). However, there was a significant reduction of Hsp60 interactions with proteins involved in amino acid and lipid metabolism with increasing temperature (p<0.05). As observed in the cytoplasmic, there was an increase in the number of interactions with partners involved in protein metabolism and modification, carbohydrate metabolism, proteassome components, and other chaperonin-like proteins in the cell wall fractions. Temperature elevation also significantly increased the number of miscellaneous proteins.

The total Hsp60 interacting proteins identified in cytoplasmic and cell wall fractions according to their specific temperature conditions are shown in Tables S1 and S2, respectively. Notably, the majority of these proteins were previously described in the proteomic analysis of extracellular vesicles produced by Hc, including proteins involved in all the metabolic processes considered [35].

### Stoichiometry of constitutive Hsp60 interactions reveals additional temperature dependent functions

Additional analysis of the constitutive proteins identified among all the temperature conditions were performed in order to validate our data and evaluate potential properties of Hsp60. According to previously described methodologies, emPAI values and spectral counts are two acceptable parameters for correlating protein concentrations in cellular extracts [39], [40]. In our model, both parameters correlated positively for the amount of proteins identified in both cytoplasm (Pearson R = 0.59, p = 0.045; Figure S1) and cell wall (Pearson R = 0.41, p<0.0001, Figure S1) subcellular fractions for the conditions tested. The percentages of interactions with Hsp60 were obtained by normalizing the emPAI values obtained for the Hsp60 interactors in each temperature conditions and cellular fractions by the sum of all emPAIs values for each condition. Values obtained display the percentage of each identified Hsp60 interacting protein depending on temperature conditions (Table 3). Results illustrate that for the majority of common proteins identified, there were no difference in terms of percentage of interactions, suggesting that most constitutive interactions occur at similar levels, independent of temperature. However, higher/lower percentages of interactions were observed (p<0.05). For example, under stress conditions, Hsp60 interactions with H2B were significantly reduced (11.42% versus 2.81 and 2.20% for 30°C compared with 37 and 37/40°C; Table 3 and Figure S2) in the cytoplasm. The percentage of Hsp60 interacting with Hsp70 was also reduced (10.74% versus 1.31 and 0.41%, respectively; Table 3 and Figure S2).

**Table 3:** Stoichiometry of constitutive Hsp60 interactions in cytoplasm and cell wall.

		Subcellular fraction					
		Cytoplasm			Cell wall		
		30	37	37/40	30	37	37/40
*Protein metabolism and modification*							
HCAG_04485	peptidylprolyl isomerase				3.52	3.12	3.56
HCAG_05988	elongation factor 2				*2.22*	*1.52*	*1.14*
HCAG_08798	translation elongation factor 1-alpha				*7.22*	*4.93*	*3.32*
*Carbohydrate metabolism*							
HCAG_00010	fructose 1_6-biphosphate aldolase				6.62	7.00	5.23
HCAG_03969	malate dehydrogenase				7.68	11.69	11.78
HCAG_04910	glyceraldehyde-3-phosphate dehydrogenase				**4.81** a	**7.97**	**9.12**
HCAG_05266	aconitase				**1.01**	**1.70**	**1.81**
HCAG_06901	malate dehydrogenase				3.89	3.96	2.44
*Nuclear*							
HCAG_03525	histone H2b	*11.42* b	*2.81*	*2.20*	7.22	9.87	4.53
HCAG_00683	woronin body major protein				*5.30*	*2.91*	*2.07*
*Chaperone-like*							
HCAG_00806	heat shock protein SSB1				2.69	3.14	3.32
HCAG_01398	hsp70-like protein	*10.74*	*1.31*	*0.41*	**5.15**	**12.54**	**11.50**
HCAG_04686	ATP-dependent molecular chaperone HSC82	1.78	1.41	2.20	4.85	5.65	3.91
HCAG_08176	heat shock protein SSC1				**1.09**	**3.11**	**4.50**
*Ribosomal*							
HCAG_02704	40S ribosomal protein S15				*9.60*	*4.52*	*6.01*
HCAG_04418	40S ribosomal protein S24				15.52	6.02	15.34
*Miscellaneous*							
HCAG_02813	ATP synthase subunit alpha				1.72	1.18	2.66
HCAG_04173	3 family protein				*3.64*	*2.49*	*1.65*
HCAG_06944	mitochondrial ATP synthase	4.85	3.49	3.98	6.23	6.66	6.11

a Bold type represents a significant (p<0.05) percentage increase with increasing temperature;

b Italics represents a significant (p<0.05) percentage decrease with increasing temperature.

At the cell wall, however, higher percentages of interactions were observed over the increasing temperature conditions (Table 3 and Figure S3). For example, comparing interactions at 30°C with 37 and 37/40°C, increased interactions occurred with enzymes involved in carbohydrate metabolism, such as, glyceraldehydes 3-phosphate dehydrogenase (4.81% versus 7.97 and 9.12%) and aconitase (1.01% versus 1.70 and 1.81%); and with chaperonins, such as Hsp70 (5.15% versus 12.54% and 11.50 heat shock protein SSC1 (1.09% versus 3.11% and 4.50%). Reductions in interactions when comparing 30°C with 37 and 37/40°C occurred with enzymes involved in protein metabolism and modification, such as elongation factor 2 (2.22 versus 1.52 and 1.14%, respectively) and translation elongation factor 1-alpha (7.22 versus 4.93 and 3.32%); nuclear proteins such as woronin body major protein (5.30 versus 2.91 and 2.07%) and ribosomal proteins such as 40S ribosomal protein S15 (9.60 versus 4.52 and 6.01%).

## Discussion

Inducible Hsps are a pool of proteins that display changes in expression in response to stress, especially to changes in temperature, alterations in pH and oxidative stress [42].Chaperones typically exert their function without structural stereo-specificity for their substrates and, despite their diversity of operating mechanisms, they can cooperate and interchange function [4]. Hsps share common functional domains, such as multiple hydrophobic peptide-binding domains with broad specificity, which bind exposed hydrophobic residues of misfolded substrate proteins, and an adenine nucleotide binding domain, which binds and hydrolyzes ATP, inducing major conformational changes on the protein resulting in folding of the substrate polypeptide from the hydrophilic chamber [43], [44].

Hsps are abundant within the cell and are located in different compartments, such as in the mitochondria, chloroplasts, endoplasmic reticulum and nucleus [45]. These proteins may display different physiological functions depending on cellular distribution. Also, the appearance of stress glycoproteins after heat shock in various subcellular fractions and modification on the intracellular distribution may occur by several mechanisms [46], [47]. Intracellular Hsps mainly play protective roles, such as facilitating protein renaturation and protein stabilization by blocking irreversible transition states [48]. These proteins are also released to the extracellular milieu by stressed cells, pointing to a potential role of these proteins as intercellular signaling molecules [49], [50], [51].

A unique feature of the Hsp60 of the fungus Hc is that it is also found on the surface of the organism [7], but its function in this subcellular location is poorly understood. Interestingly, Hc Hsp60 is the ligand recognized by the integrin CR3 (CD11b/CD18) expressed on the surface of macrophage/monocytes [7], [8]. Engagement through Hsp60 is followed by Hc internalization and inhibition of respiratory burst [29], [52], [53]. This process facilitates the capacity of the pathogen to survive and replicate within host cells [52], [54].

The ability to grow at 37°C is a crucial virulence factor in invasive human pathogens. In certain fungi, the shift in temperature from environmental temperatures to 37°C is associated with intense morphological changes resulting in a mycelia to yeast transition. Moreover, this heat-induced phenomena is accompanied by a heat shock response, which in turn results in changes in several different metabolic processes [55], [56]. Hc highly expresses Hsp60 when undergoing transition from mycelium-to-yeast [22] and, additionally, Hsp60 expression levels are strain and temperature dependent, with an expression peak between 34 and 37°C [57]. Other Hc Hsps, such as Hsp70 and Hsp82, display a similar expression pattern [58], [59]. To date, thermotolerance of Hc strains has been characterized only at the level of expression of specific morphologic phase genes, such as yps-3 [60] and heat shock proteins [59], [61]. Thermotolerance also has been correlated to the expression of the enzyme Δ-9-desaturase and temperature susceptible strains display high expression levels resulting in increase in the saturated to unsaturated fatty acid ratio of the cell membrane and higher permeability [55].

Our results show that Hsp60 levels increase in response to temperature stress in both cytoplasm and cell wall subcellular fractions. However, the magnitude of change in Hsp60 in the cell wall was less variable suggesting that in the conditions tested Hsp60 had a constitutive and regulatory function in the cell, orchestrating traffic of proteins to the cell surface. Furthermore, it suggests that Hsp60 is present at the cell wall at levels close to saturation, independent of the overall expression in the cell.

Several approaches have been applied to dissect cellular chaperonin functions in order to fully understand the protein interaction networks of cells under different conditions. Hsps have been studied in *S. cerevisiae*, revealing clear distinctions between chaperones that are functionally promiscuous and chaperones that are functionally specific [62], [63]. Furthermore, the studies have suggested the presence of endogenous multicomponent chaperones [64]. However, the scientific investigation of chaperones in other fungal species is still at an early stage.

Our data provides the first view of the Hsp60 chaperone interaction network of a dimorphic organism. The Hc Hsp60 interactome network is constructed based on Hsp60's physical protein interactions as a consequence of temperature and subcellular localization. In most cases, these interactions reflect the binding between a given chaperone and a protein complex, rather than a direct binary interaction. Hc Hsp60 interacts with a total of 58 unique proteins at 30°C, with 126 unique proteins at 37°C and 146 unique proteins at 37°C followed by treatment at 40°C. Differential interactions have been dissected in both cytoplasmic and cell wall fractions, and we identified common and unique interactions within each subcellular compartment.

Hc Hsp60 interacts with essential and non-essential proteins, suggesting a network formation wherein this protein appears to contribute significantly to the stress response. The interactome reveals that Hc Hsp60 engages nuclear chaperones, small chaperones and Hsp90 families. Hsp70 is a putative chaperone secreted by the fungus to the extracellular milieu, probably within vesicles [35], but also found on the cellular surface. Hsp70 synthesis increases soon after heat shock [57], [65] and we demonstrated more interactions of Hsp60 with Hsp70 at elevated temperatures. Thus, Hc Hsp60 possesses a promiscuous function, in various cellular compartments, and communicates with other chaperones of the cytoplasm/nucleus.

Temperature also increases the general number of interactions of the Hsp60 with proteins related to energetic metabolism, such as proteins involved in amino acid and protein metabolism, carbohydrate metabolism and fatty acid metabolism. This is accompanied by an increase in the number of interactions with proteins involved in protein and carbohydrate metabolism, specifically at the cell wall level. These increased interactions might occur in the stress recovery phase, in response to the uncoupling of oxidative phosphorylation [66] and a decline in intracellular ATP levels [67]. Additionally, it has been shown that respiration is coupled in the yeast phase at 37°C, and this change results in cellular adaptation to higher temperatures [65].

Common Hsp60-protein interactions observed under each condition evaluated have revealed quantitative differences (emPAI) depending on temperature. In the cytoplasm, a lower proportion of Hsp60 interacts with Hsp70 at higher temperatures. However, temperature increases interactions between Hsp60 and Hsp70 in the cell wall. Furthermore, cell wall Hsp60 more broadly interacts with enzymes related to carbohydrate metabolism, suggesting a trafficking function of the Hsp60 related to enhanced energy acquisition under stress conditions. H2B and M antigen also interact differentially with Hsp60 depending on temperature. A lower proportion of Hsp60 interacts with H2B in the cytoplasm and M antigen in the cell wall of yeast at higher temperatures, consistent with the function of the two proteins.

Our comprehensive analysis of Hc Hsp60 physical interactions network provides evidence for the involvement of Hsp60 in protein folding and translocation pathways. Translocation of stress may also be accomplished through the help of other chaperones, such as Hsp70. As a molecular chaperone, HSp60 appears to have diverse effects on molecular surveillance functions in order to maintain the proper function and homeostasis of multiple cellular pathways under stress conditions. Our results share similarities with the findings of studies investigating interactions among chaperones and other proteins in the model yeast *S. cereviseae* [62], [63], [64]. For example, both fungi demonstrated interactions of Hsp60 with chaperonins Hsp82 (HCAG_04686), Ssa4 (HCAG_05805, heat shock 70 kDa protein C precursor) and Sse1 (HCAG_00783, Hsp88-like protein). Furthermore, other well characterized interactions in *S. cereviseae* were observed in Hc at elevated temperatures, such as with proteins involved in carbohydrate metabolism (HCAG_05090, citrate lyase) and protein metabolism (HCAG_04297, aspartyl aminopeptidase; and HCAG_08833, peptidyl-prolylcis-trans somerase).

We conclude that Hc Hsp60 is a key regulator of diverse cellular processes, including amino acid, protein, lipid, and carbohydrate metabolism, cell signaling, replication, and expression of virulence associated proteins. Hsp60 apparently contributes with cell wall changes that allow the pathogen to survive under stress conditions [16]. In addition, these data open a new perspective since these interactions could potentially modify the way that host immune cells recognize the pathogen possibly modulating the immune response.

Due to the high homology of Hsp60 proteins in different organisms, the fact that Hsp60 is secreted by Hc [35], and our present demonstration of the promiscuity of Hc Hsp60, this protein could also act as a scavenger of host proteins and thus modify host immune responses. The broad interaction capacity of Hsp60 opens numerous interesting avenues for future study, including drug targeting and immunoterapy for treating life-threatening fungal infections [6].

## Supporting Information

Table S1 Cytoplasm Hsp60 interactome under different temperature stress conditions.
(0.21 MB DOC)

Table S2 Cell wall Hsp60 interactome under different temperature stress conditions.
(0.16 MB DOC)

Figure S1 Correlations of the two parameters used to quantitatively evaluate the mass spectrometry analyses, emPAI and spectral counts. Correlation of emPAI and spectral counts for all of the proteins identified in the (A) cytoplasmic fraction and (B) cell wall fraction.
(0.67 MB TIF)

Figure S2 Graphic representation of the levels of interaction of Hsp60 with distinct interaction partners in the cytoplasm at different temperature conditions. Results illustrate that differences were observed for the majority of common proteins identified, as shown in Table 3.
(0.35 MB TIF)

Figure S3 Graphic representation of levels of interaction of the Hsp60 with distinct interaction partners in the cell wall at different temperature conditions. As in Table 3, the results illustrate that there were no difference in terms of percentage of interactions for the majority of common proteins identified, suggesting that in most cases constitutive interactions occur at similar levels, independent of temperature.
(1.35 MB TIF)

## References

[1] Lindquist S (1986). The heat-shock response.. Annu Rev Biochem.

[2] Wu C (1995). Heat shock transcription factors: structure and regulation.. Annu Rev Cell Dev Biol.

[3] Santoro MG (2000). Heat shock factors and the control of the stress response.. Biochem Pharmacol.

[4] Saibil HR (2008). Chaperone machines in action.. Curr Opin Struct Biol.

[5] Li Z, Srivastava P (2004). Heat-shock proteins.. Curr Protoc Immunol Appendix 1: Appendix.

[6] Burnie JP, Carter TL, Hodgetts SJ, Matthews RC (2006). Fungal heat-shock proteins in human disease.. FEMS Microbiol Rev.

[7] Long KH, Gomez FJ, Morris RE, Newman SL (2003). Identification of heat shock protein 60 as the ligand on *Histoplasma capsulatum* that mediates binding to CD18 receptors on human macrophages.. J Immunol.

[8] Habich C, Kempe K, Gomez FJ, Lillicrap M, Gaston H (2006). Heat shock protein 60: identification of specific epitopes for binding to primary macrophages.. FEBS Lett.

[9] Guimaraes AJ, Frases S, Gomez FJ, Zancope-Oliveira RM, Nosanchuk JD (2009). Monoclonal antibodies to heat shock protein 60 alter the pathogenesis of *Histoplasma capsulatum*.. Infect Immun.

[10] Guimaraes AJ, Frases S, Pontes B, MD DEC, Rodrigues ML (2010). Agglutination of *Histoplasma capsulatum* by IgG monoclonal antibodies against Hsp60 impacts macrophage effector functions.. Infect Immun.

[11] Gomez BL, Figueroa JI, Hamilton AJ, Ortiz BL, Robledo MA (1997). Development of a novel antigen detection test for histoplasmosis.. J Clin Microbiol.

[12] Gomez FJ, Gomez AM, Deepe GS (1992). An 80-kilodalton antigen from *Histoplasma capsulatum* that has homology to heat shock protein 70 induces cell-mediated immune responses and protection in mice.. Infect Immun.

[13] Lopes LC, Guimaraes AJ, de Cerqueira MD, Gomez BL, Nosanchuk JD (2010). A *Histoplasma capsulatum*-specific IgG1 isotype monoclonal antibody, H1C, to a 70-kilodalton cell surface protein is not protective in murine histoplasmosis.. Clin Vaccine Immunol.

[14] Shapiro RS, Cowen L Coupling temperature sensing and development: Hsp90 regulates morphogenetic signalling in *Candida albicans*.. Virulence.

[15] Shapiro RS, Uppuluri P, Zaas AK, Collins C, Senn H (2009). Hsp90 orchestrates temperature-dependent *Candida albicans* morphogenesis via Ras1-PKA signaling.. Curr Biol.

[16] Shaner L, Gibney PA, Morano KA (2008). The Hsp110 protein chaperone Sse1 is required for yeast cell wall integrity and morphogenesis.. Curr Genet.

[17] Gophna U, Ron EZ (2003). Virulence and the heat shock response.. Int J Med Microbiol.

[18] Cabiscol E, Belli G, Tamarit J, Echave P, Herrero E (2002). Mitochondrial Hsp60, resistance to oxidative stress, and the labile iron pool are closely connected in *Saccharomyces cerevisiae*.. J Biol Chem.

[19] Voos W (2003). A new connection: chaperones meet a mitochondrial receptor.. Mol Cell.

[20] Voos W (2009). Mitochondrial protein homeostasis: the cooperative roles of chaperones and proteases.. Res Microbiol.

[21] Voos W, Rottgers K (2002). Molecular chaperones as essential mediators of mitochondrial biogenesis.. Biochim Biophys Acta.

[22] Kamei K, Brummer E, Clemons KV, Stevens DA (1992). Induction of stress protein synthesis in *Histoplasma capsulatum* by heat, low pH and hydrogen peroxide.. J Med Vet Mycol.

[23] Allendoerfer R, Biovin GP, Deepe GS (1997). Modulation of immune responses in murine pulmonary histoplasmosis.. J Infect Dis.

[24] Pitarch A, Sanchez M, Nombela C, Gil C (2002). Sequential fractionation and two-dimensional gel analysis unravels the complexity of the dimorphic fungus *Candida albicans* cell wall proteome.. Mol Cell Proteomics.

[25] Mrsa V, Seidl T, Gentzsch M, Tanner W (1997). Specific labelling of cell wall proteins by biotinylation. Identification of four covalently linked O-mannosylated proteins of *Saccharomyces cerevisiae*.. Yeast.

[26] Nosanchuk JD, Steenbergen JN, Shi L, Deepe GS, Casadevall A (2003). Antibodies to a cell surface histone-like protein protect against *Histoplasma capsulatum*.. J Clin Invest.

[27] Bradford MM (1976). A rapid and sensitive method for the quantitation of microgram quantities of protein utilizing the principle of protein-dye binding.. Anal Biochem.

[28] Pizzini CV, Zancope-Oliveira RM, Reiss E, Hajjeh R, Kaufman L (1999). Evaluation of a western blot test in an outbreak of acute pulmonary histoplasmosis.. Clin Diagn Lab Immunol.

[29] Guimaraes AJ, Hamilton AJ, de MGHL, Nosanchuk JD, Zancope-Oliveira RM (2008). Biological function and molecular mapping of M antigen in yeast phase of *Histoplasma capsulatum*.. PLoS One.

[30] Guimaraes AJ, Almeida MA, Pizzini CV, Peralta JM, Nosanchuk JD (2010). Evaluation of an Enzyme Linked Immunosorbent Assay (ELISA) using purified, deglycosylated histoplasmin for different clinical manifestations of Histoplasmosis.. Microbiology Research.

[31] Gomez FJ, Allendoerfer R, Deepe GS (1995). Vaccination with recombinant heat shock protein 60 from Histoplasma capsulatum protects mice against pulmonary histoplasmosis.. Infect Immun.

[32] Stone KL, Williams KR (1996). Enzymatic Digestion of Proteins in Solution and in SDS Polyacrylamide Gels..

[33] Jurado JD, Rael ED, Lieb CS, Nakayasu E, Hayes WK (2007). Complement inactivating proteins and intraspecies venom variation in *Crotalus oreganus* helleri.. Toxicon.

[34] Lundgren DH, Han DK, Eng JK (2005). Protein identification using TurboSEQUEST.. Curr Protoc Bioinformatics.

[35] Albuquerque PC, Nakayasu ES, Rodrigues ML, Frases S, Casadevall A (2008). Vesicular transport in *Histoplasma capsulatum*: an effective mechanism for trans-cell wall transfer of proteins and lipids in ascomycetes.. Cell Microbiol.

[36] Mewes HW, Frishman D, Mayer KF, Munsterkotter M, Noubibou O (2006). MIPS: analysis and annotation of proteins from whole genomes in 2005.. Nucleic Acids Res.

[37] Stark C, Breitkreutz BJ, Reguly T, Boucher L, Breitkreutz A (2006). BioGRID: a general repository for interaction datasets.. Nucleic Acids Res.

[38] Krogan NJ, Cagney G, Yu H, Zhong G, Guo X (2006). Global landscape of protein complexes in the yeast *Saccharomyces cerevisiae*.. Nature.

[39] Liu H, Sadygov RG, Yates JR (2004). A model for random sampling and estimation of relative protein abundance in shotgun proteomics.. Anal Chem.

[40] Ishihama Y, Oda Y, Tabata T, Sato T, Nagasu T (2005). Exponentially modified protein abundance index (emPAI) for estimation of absolute protein amount in proteomics by the number of sequenced peptides per protein.. Mol Cell Proteomics.

[41] Gomez FJ, Gomez AM, Deepe GS (1991). Protective efficacy of a 62-kilodalton antigen, HIS-62, from the cell wall and cell membrane of *Histoplasma capsulatum* yeast cells.. Infect Immun.

[42] Kaufmann SH (1990). Heat-shock proteins: a missing link in the host-parasite relationship?. Med Microbiol Immunol.

[43] Elad N, Farr GW, Clare DK, Orlova EV, Horwich AL (2007). Topologies of a substrate protein bound to the chaperonin GroEL.. Mol Cell.

[44] Horwich AL, Fenton WA, Chapman E, Farr GW (2007). Two families of chaperonin: physiology and mechanism.. Annu Rev Cell Dev Biol.

[45] Habich C, Burkart V (2007). Heat shock protein 60: regulatory role on innate immune cells.. Cell Mol Life Sci.

[46] Jethmalani SM, Henle KJ, Gazitt Y, Walker PD, Wang SY (1997). Intracellular distribution of heat-induced stress glycoproteins.. J Cell Biochem.

[47] Soltys BJ, Gupta RS (1997). Cell surface localization of the 60 kDa heat shock chaperonin protein (hsp60) in mammalian cells.. Cell Biol Int.

[48] Jethmalani SM, Henle KJ (1997). Intracellular distribution of stress glycoproteins in a heat-resistant cell model expressing human HSP70.. Biochem Biophys Res Commun.

[49] Barreto A, Gonzalez JM, Kabingu E, Asea A, Fiorentino S (2003). Stress-induced release of HSC70 from human tumors.. Cell Immunol.

[50] Davies EL, Bacelar MM, Marshall MJ, Johnson E, Wardle TD (2006). Heat shock proteins form part of a danger signal cascade in response to lipopolysaccharide and GroEL.. Clin Exp Immunol.

[51] Hunter-Lavin C, Davies EL, Bacelar MM, Marshall MJ, Andrew SM (2004). Hsp70 release from peripheral blood mononuclear cells.. Biochem Biophys Res Commun.

[52] Eissenberg LG, Goldman WE (1987). Histoplasma capsulatum fails to trigger release of superoxide from macrophages.. Infect Immun.

[53] Wolf AM (1987). Histoplasma capsulatum osteomyelitis in the cat.. J Vet Intern Med.

[54] Wolf JE, Kerchberger V, Kobayashi GS, Little JR (1987). Modulation of the macrophage oxidative burst by Histoplasma capsulatum.. J Immunol.

[55] Maresca B, Kobayashi G (1993). Changes in membrane fluidity modulate heat shock gene expression and produced attenuated strains in the dimorphic fungus *Histoplasma capsulatum*.. Arch Med Res.

[56] Maresca B, Kobayashi GS (1989). Dimorphism in *Histoplasma capsulatum*: a model for the study of cell differentiation in pathogenic fungi.. Microbiol Rev.

[57] Shearer G, Birge CH, Yuckenberg PD, Kobayashi GS, Medoff G (1987). Heat-shock proteins induced during the mycelial-to-yeast transitions of strains of *Histoplasma capsulatum*.. J Gen Microbiol.

[58] Minchiotti G, Gargano S, Maresca B (1992). Molecular cloning and expression of hsp82 gene of the dimorphic pathogenic fungus *Histoplasma capsulatum*.. Biochim Biophys Acta.

[59] Caruso M, Sacco M, Medoff G, Maresca B (1987). Heat shock 70 gene is differentially expressed in *Histoplasma capsulatum* strains with different levels of thermotolerance and pathogenicity.. Mol Microbiol.

[60] Keath EJ, Painter AA, Kobayashi GS, Medoff G (1989). Variable expression of a yeast-phase-specific gene in *Histoplasma capsulatum* strains differing in thermotolerance and virulence.. Infect Immun.

[61] Patriarca EJ, Kobayashi GS, Maresca B (1992). Mitochondrial activity and heat-shock response during morphogenesis in the pathogenic fungus *Histoplasma capsulatum*.. Biochem Cell Biol.

[62] Tapley TL, Franzmann TM, Chakraborty S, Jakob U, Bardwell JC Protein refolding by pH-triggered chaperone binding and release. Proc Natl Acad Sci U S A.

[63] Tapley TL, Korner JL, Barge MT, Hupfeld J, Schauerte JA (2009). Structural plasticity of an acid-activated chaperone allows promiscuous substrate binding.. Proc Natl Acad Sci U S A.

[64] Gong Y, Kakihara Y, Krogan N, Greenblatt J, Emili A (2009). An atlas of chaperone-protein interactions in *Saccharomyces cerevisiae*: implications to protein folding pathways in the cell.. Mol Syst Biol.

[65] Lambowitz AM, Kobayashi GS, Painter A, Medoff G (1983). Possible relationship of morphogenesis in pathogenic fungus, *Histoplasma capsulatum*, to heat shock response.. Nature.

[66] Borgia G, Tallarino A, Crowell J, Lambiase A, Cicciarello S (1990). The effect of temperature on the ultrastructure of *Histoplasma capsulatum* during the mycelium-yeast transition.. Mycoses.

[67] Maresca B, Lambowitz AM, Kumar VB, Grant GA, Kobayashi GS (1981). Role of cysteine in regulating morphogenesis and mitochondrial activity in the dimorphic fungus *Histoplasma capsulatum*.. Proc Natl Acad Sci U S A.

