# Supplementary material for: Histoplasma capsulatum Heat-Shock 60 Orchestrates the Adaptation of the Fungus to Temperature Stress
Source: PLoS One. 2011 Feb 10;6(2):e14660. doi: 10.1371/journal.pone.0014660 (PMC3037374; doi:10.1371/journal.pone.0014660)
Supplement: Table S1 — Cytoplasm Hsp60 interactome under different temperature stress conditions. (0.21 MB DOC) [file pone.0014660.s001.doc]

**Table S1**: Cytoplasm Hsp60 interactome under different temperature stress conditions.

| **ID number** | **Protein name** | **Molecular function (Gene Onthology)** |
| --- | --- | --- |
| **30, 37, 37/40^o^C** |  |  |
| *Nuclear* |  |  |
| HCAG_03525 | histone H2b | nucleosome, DNA binding, nucleosome assembly, nucleus |
| *Chaperone-like* |  |  |
| HCAG_01398 | hsp70-like protein | ATP binding, response to stress |
| HCAG_04686 | ATP-dependent molecular chaperone HSC82 | protein folding, unfolded protein binding, ATP binding, response to stress |
| *Miscellaneous* |  |  |
| HCAG_06944 | mitochondrial ATP synthase | proton-transporting ATP synthase complex, catalytic core F(1), hydrogen-exporting ATPase activity, phosphorylative mechanism, hydrogen ion transporting ATP synthase activity, rotational mechanism, ATP synthesis coupled proton transport, ATP binding, proton-transporting ATPase activity, rotational mechanism |
|  |  |  |
| **37, 37/40^o^C** |  |  |
| *Amino acid metabolism* | |  |
| HCAG_00014 | homoserine dehydrogenase | threonine biosynthetic process, NADP or NADPH binding, response to stress, methionine biosynthetic process, isoleucine biosynthetic process, oxidation reduction, transferase activity, homoserine dehydrogenase activity |
| HCAG_00676 | methylcrotonoyl-CoA carboxylase beta chain | propionyl-CoA carboxylase activity, methylcrotonoyl-CoA carboxylase activity |
| HCAG_02524 | 3-hydroxyisobutyryl-CoA hydrolase | metabolic process, hydrolase activity |
| HCAG_05565 | cobalamin-independent methionine synthase MetH/D | methionine biosynthetic process, zinc ion binding, 5-methyltetrahydropteroyltriglutamate-homocysteine S-methyltransferase activity |
| HCAG_06102 | aspartate aminotransferase | pyridoxal phosphate binding, cellular amino acid metabolic process, biosynthetic process, L-aspartate:2-oxoglutarate aminotransferase activity |
| HCAG_07004 | O-acetylhomoserine | pyridoxal phosphate binding, cysteine biosynthetic process, lyase activity, O-acetylhomoserine aminocarboxypropyltransferase activity |
| HCAG_07418 | serine hydroxymethyltransferase | pyridoxal phosphate binding, L-serine metabolic process, glycine hydroxymethyltransferase activity, one-carbon compound metabolic process, glycine metabolic process |
| HCAG_07571 | glycine dehydrogenase | pyridoxal phosphate binding, oxidation reduction, glycine metabolic process, aminomethyltransferase activity, glycine dehydrogenase (decarboxylating) activity, lyase activity |
| HCAG_08678 | aspartate aminotransferase | pyridoxal phosphate binding, cellular amino acid metabolic process, biosynthetic process, L-aspartate:2-oxoglutarate aminotransferase activity |
| HCAG_10265 | adenosylhomocysteinase | one-carbon compound metabolic process, adenosylhomocysteinase activity, binding |
| *Protein metabolism and modification* | |  |
| HCAG_04485 | peptidylprolyl isomerase | protein folding, cytoplasm, peptidyl-prolyl cis-trans isomerase activity |
| HCAG_08798 | translation elongation factor 1-alpha | cytoplasm, translation elongation factor activity, GTP binding, translational elongation, GTPase activity |
| HCAG_08833 | peptidyl-prolyl cis-trans isomerase | protein folding, cytoplasm, peptidyl-prolyl cis-trans isomerase activity, binding |
| *Carbohydrate metabolism* | |  |
| HCAG_00010 | fructose 1,6-biphosphate aldolase | glycolysis, fructose-bisphosphate aldolase activity, zinc ion binding |
| HCAG_03322 | fructose-1_6-bisphosphatase | carbohydrate metabolic process, fructose 1,6-bisphosphate 1-phosphatase activity |
| HCAG_03385 | phosphoglycerate kinase | cytoplasm, phosphoglycerate kinase activity, ATP binding, glycolysis |
| HCAG_03803 | hydroxymethylglutaryl-CoA lyase | hydroxymethylglutaryl-CoA lyase activity, metabolic process |
| HCAG_03969 | malate dehydrogenase | binding, L-malate dehydrogenase activity, malate metabolic process, oxidation reduction, tricarboxylic acid cycle, glycolysis |
| HCAG_03972 | dihydrolipoamide acetyltransferase component | pyruvate dehydrogenase complex, pyruvate metabolic process, dihydrolipoyllysine-residue acetyltransferase activity, protein binding, intracellular protein transport, lipoic acid binding, |
| HCAG_04329 | glucose-6-phosphate 1-dehydrogenase | glucose metabolic process, glucose-6-phosphate dehydrogenase activity, oxidation reduction, binding |
| HCAG_04358 | peroxisomal NADP-dependent isocitrate dehydrogenase | manganese ion binding, phosphoglycolate phosphatase activity, isocitrate dehydrogenase (NADP+) activity, oxidation reduction, magnesium ion binding, glyoxylate cyclemitochondrion, isocitrate metabolic process, tricarboxylic acid cycle, |
| HCAG_04910 | glyceraldehyde-3-phosphate dehydrogenase | cytoplasm, NAD or NADH binding, response to stress, oxidation reduction, glycolysis, glyceraldehyde-3-phosphate dehydrogenase (phosphorylating) activity, |
| HCAG_05090 | 2-methylcitrate synthase | cellular carbohydrate metabolic process, acyltransferase activity, propionate metabolic process, methylcitrate cycle, mitochondrial matrix, 2-methylcitrate synthase activity |
| HCAG_05266 | aconitase | aconitate hydratase activity, tricarboxylic acid cycle, 4 iron, 4 sulfur cluster binding |
| HCAG_05884 | 6-phosphogluconate dehydrogenase | NADP or NADPH binding, oxidation reduction, pentose-phosphate shunt, phosphogluconate dehydrogenase (decarboxylating) activity |
| HCAG_06981 | citrate synthase | cellular carbohydrate metabolic process, acyltransferase activity, mitochondrial matrix, citrate (Si)-synthase activity, tricarboxylic acid cycle, lyase activity |
| HCAG_07781 | pyruvate kinase | pyruvate kinase activity, potassium ion binding, magnesium ion binding, glycolysis |
| *Lipid, fatty acid and steroid metabolism* | |  |
| HCAG_07725 | 3-hydroxybutyryl-CoA dehydrogenase | fatty acid metabolic process, oxidation reduction, coenzyme binding, 3-hydroxybutyryl-CoA dehydrogenase activity |
| HCAG_08621 | acetyl-CoA acetyltransferase | acetyl-CoA C-acetyltransferase activity, metabolic process |
| *Nucleoside, nucleotide and nucleic acid metabolism* | |  |
| HCAG_00544 | nucleoside-diphosphate kinase | GTP biosynthetic process, CTP biosynthetic process, nucleoside diphosphate kinase activity, mycelium development, UTP biosynthetic process, ATP binding, magnesium ion binding |
| *Cell growth/division* | |  |
| HCAG_00717 | septin-1 | septin complex, cell cycle, protein binding, GTP binding |
| HCAG_02452 | cell division cycle protein | nucleoside-triphosphatase activity, cell cycle, protein transport, ATP binding, response to stress, cell division |
| *Nuclear* | |  |
| HCAG_01311 | small nuclear ribonucleoprotein Sm D3 | nucleic acid binding, ribonucleoprotein complex, growth or development of symbiont on or near host |
| HCAG_02914 | glycine-rich protein | nucleic acid binding, mycelium development, nucleotide binding |
| HCAG_03524 | histone H2A | nucleosome, DNA binding, nucleosome assembly, nucleus, DNA repair |
| HCAG_03596 | HMG | hydrolase activity, nucleus, DNA binding |
| HCAG_03885 | histone h4 | nucleosome, mycelium development, DNA binding, nucleosome assembly, nucleus |
| HCAG_04835 | proliferating cell nuclear antigen | substrate-specific transmembrane transporter activity, DNA polymerase processivity factor activity, DNA binding, transmembrane transport, regulation of DNA replication, PCNA complex, integral to membrane, nucleus, |
| HCAG_06702 | hypothetical protein | nucleosome, DNA binding, nucleosome assembly, nucleus |
| *Cytoskeletal* | |  |
| HCAG_02068 | tubulin alpha-1 subunit | microtubule-based movement, protein complex, GTP binding, protein polymerization, structural molecule activity, GTPase activity, microtubule |
| HCAG_08288 | tubulin subunit alpha-2 | microtubule-based movement, protein complex, GTP binding, protein polymerization, structural molecule activity, GTPase activity, microtubule |
| *Plasma membrane* | |  |
| HCAG_06977 | Plasma membrane ATPase | hydrogen-exporting ATPase activity, phosphorylative mechanism, integral to membrane, ATP biosynthetic process, ATP binding, magnesium ion binding, proton transport, plasma membrane |
| *Anti-oxidant* | |  |
| HCAG_01543 | Superoxide dismutase | oxidation reduction, metal ion binding, superoxide metabolic process, superoxide dismutase activity |
| HCAG_03448 | manganese superoxide dismutase | metal ion binding, mycelium development, superoxide dismutase activity, oxidation reduction, superoxide metabolic process, mitochondrion |
| HCAG_06210 | thiol-specific antioxidant | cell redox homeostasis, oxidoreductase activity, antioxidant activity, phospholipase A2 activity |
| HCAG_08064 | catalase B | heme binding, oxidation reduction, catalase activity, hydrogen peroxide catabolic process |
| *Proteasome component* | |  |
| HCAG_00053 | proteasome subunit alpha | proteasome core complex, ubiquitin-dependent protein catabolic process, cytosol, threonine-type endopeptidase activity |
| HCAG_00347 | proteasome component | proteasome core complex, ubiquitin-dependent protein catabolic process, nucleus, cytosol, threonine-type endopeptidase activity |
| HCAG_03939 | proteasome component | proteasome core complex, ubiquitin-dependent protein catabolic process, cytosol, threonine-type endopeptidase activity |
| HCAG_04090 | proteasome component | proteasome core complex, ubiquitin-dependent protein catabolic process, cytosol, threonine-type endopeptidase activity |
| HCAG_04101 | proteasome component | proteasome core complex, ubiquitin-dependent protein catabolic process, cytosol, threonine-type endopeptidase activity |
| HCAG_04198 | proteasome component | proteasome core complex, ubiquitin-dependent protein catabolic process, nucleus, cytosol, threonine-type endopeptidase activity |
| HCAG_06342 | proteasome component | proteasome core complex, ubiquitin-dependent protein catabolic process, nucleus, cytosol, threonine-type endopeptidase activity |
| HCAG_08215 | proteasome subunit alpha | proteasome core complex, ubiquitin-dependent protein catabolic process, cytosol, threonine-type endopeptidase activity |
| *Chaperone-like* | |  |
| HCAG_04943 | hsp10-like protein | protein folding, ATP binding, response to stress |
| HCAG_05805 | heat shock 70 kDa protein C precursor | endoplasmic reticulum lumen, secretion by cell, oxidation reduction, response to unfolded protein, ATP binding, endoplasmic reticulum, 2-alkenal reductase activity |
| HCAG_08176 | heat shock protein SSC1 | protein folding, endonuclease activity, response to stress, oxidation reduction, ATP binding, unfolded protein binding, mitochondrion, 2-alkenal reductase activity |
| *Ribosomal* | |  |
| HCAG_01850 | 60S ribosomal protein L1 | ribosome, structural constituent of ribosome, RNA binding, translation, RNA processing |
| HCAG_01947 | ribosomal protein S23 | structural constituent of ribosome, small ribosomal subunit, translation |
| HCAG_02703 | 60S acidic ribosomal protein P2 | structural constituent of ribosome, small ribosomal subunit, translation |
| HCAG_04987 | ribosomal protein L22e | ribosome, structural constituent of ribosome, translation |
| *Miscellaneous* | |  |
| HCAG_00437 | cytochrome c oxidase chain VI | cytochrome-c oxidase activity |
| HCAG_01212 | sulfate adenylyltransferase | cytoplasm, kinase activity, sulfate adenylyltransferase (ATP) activity, methionine biosynthetic process, ATP binding, cysteine biosynthetic process, sulfate assimilation |
| HCAG_02813 | ATP synthase subunit alpha | proton-transporting ATP synthase complex, catalytic core F(1), hydrogen ion transporting ATP synthase activity (rotational mechanism), mycelium development, ATP synthesis coupled proton transport, ATP binding, proton-transporting ATPase activity (rotational mechanism) |
| HCAG_02994 | pyridoxine biosynthesis protein pyroA [validated] | pyridoxine biosynthetic process, lyase activity |
| HCAG_03323 | fumarate reductase flavoprotein subunit | heme binding, oxidation reduction, succinate dehydrogenase activity, electron carrier activity, fumarate reductase (NADH) activity |
| HCAG_04173 | 14-3-3 family protein | protein domain specific binding |
| HCAG_04527 | 14-3-3 family protein ArtA | protein domain specific binding |
| HCAG_04799 | ATP synthase gamma chain | proton-transporting ATP synthase complex (catalytic core F(1)), hydrogen ion transporting ATP synthase activity (rotational mechanism), ATP synthesis coupled, proton-transporting ATPase activity (rotational mechanism)growth or development of symbiont on or near hostproton transport |
| HCAG_04999 | spermidine synthase | spermidine synthase activity |
| HCAG_05099 | nascent polypeptide-associated complex subunit alpha | cytoplasm, nucleus, regulation of transcription, DNA-dependent, protein transport |
| HCAG_06005 | acetolactate synthase | two-component sensor activity, peptidyl-histidine phosphorylation, ATP binding, magnesium ion binding, signal transduction, thiamin pyrophosphate binding, lyase activity |
| HCAG_07206 | glutathione-dependent formaldehyde dehydrogenase | oxidation reduction, alcohol dehydrogenase activity, S-(hydroxymethyl)glutathione dehydrogenase activity, zinc ion binding, ethanol oxidation |
| HCAG_08058 | enoyl-CoA hydratase/isomerase family protein | metabolic process, enoyl-CoA hydratase activity, isomerase activity, 3-hydroxybutyryl-CoA dehydratase activity |
| HCAG_08367 | aldehyde dehydrogenase | oxidation reduction, aldehyde dehydrogenase (NAD) activity |
| HCAG_08561 | alcohol dehydrogenase I | cytoplasm, oxidation reduction, alcohol dehydrogenase activity, zinc ion binding |
| HCAG_08720 | mannitol-1-phosphate dehydrogenase | oxidation reduction, mannitol-1-phosphate 5-dehydrogenase activity, coenzyme binding, D-iditol 2-dehydrogenase activity |
| HCAG_08825 | dihydrolipoamide dehydrogenase | cell redox homeostasis, FAD binding, cytoplasm, oxidation reduction, electron carrier activity, dihydrolipoyl dehydrogenase activity |
| HCAG_10553 | carbonic anhydrase family protein | intracellular, growth or development of symbiont on or near host, zinc ion binding, carbonate dehydratase activity, carbon utilization |
|  |  |  |
| **30^o^C** |  |  |
| *Cell signaling* | |  |
| HCAG_06941 | GTP-binding protein | GTP binding, aminopeptidase activity, zinc ion binding, metallopeptidase activity, small GTPase mediated signal transduction, protein transport |
| *Cytoskeletal* | |  |
| HCAG_08210 | actin | cytoskeleton, cytoplasm, ATP binding, protein binding |
| *Ribosomal* | |  |
| HCAG_04418 | 40S ribosomal protein S24 | ribosome, structural constituent of ribosome, translation, nucleotide binding |
| *Miscellaneous* | |  |
| HCAG_06678 | Vesicle-fusing ATPase | ATP binding, nucleoside-triphosphatase activity |
| *Biological process unclassified* | |  |
| HCAG_04741 | conserved hypothetical protein | undefined |
| HCAG_05989 | conserved hypothetical protein | undefined |
|  |  |  |
| **37^o^C** |  |  |
| *Amino acid metabolism* | |  |
| HCAG_02710 | cystathionine gamma-lyase | pyridoxal phosphate binding, cellular amino acid metabolic process, lyase activity |
| HCAG_03751 | aspartate aminotransferase | aspartate aminotransferase, aspartate aminotransferase, aspartate aminotransferase, aspartate aminotransferase, cellular amino acid metabolic process, biosynthetic process, L-aspartate:2-oxoglutarate aminotransferase activity |
| HCAG_08086 | kynureninase | NAD biosynthetic process, kynureninase activity, tryptophan catabolic process, cytoplasm, pyridoxal phosphate binding |
| *Protein metabolism and modification* | |  |
| HCAG_01178 | translation initiation factor 3 | translational initiation, translation initiation factor activity |
| HCAG_01784 | elongation factor 1-beta | translation elongation factor activity, eukaryotic translation elongation factor 1 complex, translational elongation |
| HCAG_03630 | proteindisulfidisomerase | endoplasmic reticulum lumen, cell redox homeostasis, calcium ion binding, protein disulfide isomerase activity, endoplasmic reticulum |
| HCAG_04297 | aspartyl aminopeptidase | vacuole, aminopeptidase activity, proteolysis, zinc ion binding, metallopeptidase activity, aminopeptidase activity, proteolysis, zinc ion binding, metallopeptidase activity |
| HCAG_07345 | peptidyl-prolyl cis/trans isomerase | protein folding, peptidyl-prolyl cis-trans isomerase activity, protein binding |
| *Nucleoside, nucleotide and nucleic acid metabolism* | |  |
| HCAG_06908 | purine nucleoside phosphorylase | purine-nucleoside phosphorylase activity, cytosol, purine nucleoside catabolic process, nucleus |
| *Nuclear* | |  |
| HCAG_04745 | Y20 protein | oxidoreductase activity, FMN binding, oxidation reduction, negative regulation of transcription |
| *Cell wall architecture* | |  |
| HCAG_04277 | alpha-mannosidase | mannose metabolic process, alpha-mannosidase activity, zinc ion binding |
| *Plasma membrane* | |  |
| HCAG_03815 | ATP synthase subunit 5 | proton-transporting ATP synthase complex (catalytic core F(1)) hydrogen ion transporting ATP synthase activity (rotational mechanism), ATP synthesis coupled proton transport, proton-transporting ATPase activity (rotational mechanism) |
| *Proteasome component* | |  |
| HCAG_05739 | proteasome component | proteasome core complex, ubiquitin-dependent protein catabolic process, nucleus, cytosol, threonine-type endopeptidase activity |
| HCAG_05910 | proteasome component | proteasome core complex, ubiquitin-dependent protein catabolic process, nucleus, cytosol, threonine-type endopeptidase activity |
| *Ribosomal* | |  |
| HCAG_08515 | 60S ribosomal protein L2 | ribosome, structural constituent of ribosome, translation |
| *Miscellaneous* |  |  |
| HCAG_00301 | predicted protein | cytochrome p450 55a3, cytochrome p450 55a3, cytochrome p450 55a3, cytochrome p450 55a3, cytochrome p450 55a3, cytochrome p450 55a3, cytochrome p450 55a3, electron carrier activity, monooxygenase activity, heme binding, transcription, nucleus, zinc ion binding |
| HCAG_01404 | coproporphyrinogen III oxidase | oxidation reduction, coproporphyrinogen oxidase activity, porphyrin biosynthetic process |
| HCAG_01918 | glutaminase A | hydrolase activity |
| HCAG_02027 | quinone oxidoreductase | acyltransferase activity, oxidation reduction, mycocerosate synthase activity, zinc ion binding, NADPH:quinone reductase activity |
| HCAG_02570 | cytochrome P450 | heme binding, monooxygenase activity, electron carrier activity |
| HCAG_08725 | NADH-ubiquinone oxidoreductase | mitochondrial inner membrane, NADH dehydrogenase (ubiquinone) activity, respiratory electron transport chain |
| HCAG_08778 | pyruvate decarboxylase | thiamin pyrophosphate binding, pyruvate decarboxylase activity, magnesium ion binding |
| HCAG_09033 | glutathione synthetase | ATP binding, glutathione biosynthetic process, glutathione synthase activity |
| *Biological process unclassified* | |  |
| HCAG_06110 | immunogenic protein | undefined |
| HCAG_08343 | predicted protein | undefined |
|  |  |  |
| **40^o^C** |  |  |
| *Amino acid metabolism* | |  |
| HCAG_02120 | fumarylacetoacetase hydrolase family protein | fumarylacetoacetase activity, tyrosine catabolic process, calcium ion binding, magnesium ion binding, L-phenylalanine catabolic process |
| *Protein metabolism and modification* | |  |
| HCAG_02992 | xaa-pro aminopeptidase | proteolysis, metalloexopeptidase activity, aminopeptidase activity |
| HCAG_04206 | eukaryotic translation initiation factor 2 gamma subunit | translation elongation factor activity, GTPase activity, GTP binding |
| HCAG_05988 | hypothetical protein similar to elongation factor 2 | translation elongation factor activity, GTPase activity, GTP binding |
| HCAG_06060 | calnexin | endoplasmic reticulum membrane, sugar binding, protein folding, calcium ion binding, unfolded protein binding, integral to membrane |
| HCAG_06641 | UDP-galactopyranose mutase | UDP-galactopyranose mutase activity, oxidoreductase activity, electron carrier activity |
| *Carbohydrate metabolism* | |  |
| HCAG_06317 | Succinate dehydrogenase flavoprotein subunit | FAD binding, electron transport chain, tricarboxylic acid cycle, electron carrier activity, succinate dehydrogenase (ubiquinone) activity |
| HCAG_06901 | malate dehydrogenase | binding, L-malate dehydrogenase activity, malate metabolic process, oxidation reduction, glycolysis |
| HCAG_07697 | succinyl-CoA ligase beta-chain | metabolic process, ATP binding, succinate-CoA ligase (ADP-forming) activity, succinate-CoA ligase (GDP-forming) activity |
| HCAG_08202 | glucose-6-phosphate isomerase | cytoplasm, gluconeogenesis, glucose-6-phosphate isomerase activity, glycolysis |
| *Lipid, fatty acid and steroid metabolism* | |  |
| HCAG_00678 | 3-methylcrotonyl-CoA carboxylase biotin-containing subunit | biotin binding, methylcrotonoyl-CoA carboxylase activity, metabolic process, ATP binding |
| HCAG_01596 | 3-ketoacyl-CoA thiolase peroxisomal A | acetyl-CoA C-acyltransferase activity, acetyl-CoA C-acetyltransferase activity, metabolic process |
| HCAG_01606 | acetate-CoA ligase | acetoin catabolic process, acetate-CoA ligase activity, AMP binding |
| HCAG_05596 | diphosphomevalonate decarboxylase | UDP-3-O-[3-hydroxymyristoyl] N-acetylglucosamine deacetylase activity, diphosphomevalonate decarboxylase activity, kinase activity, phosphorylation, isoprenoid biosynthetic process, ATP binding, lipid A biosynthetic process |
| *Nucleoside, nucleotide and nucleic acid metabolism* | |  |
| HCAG_04224 | bifunctional purine biosynthesis protein ADE16 | phosphoribosylaminoimidazolecarboxamide formyltransferase activity, IMP cyclohydrolase activity, IMP biosynthetic process |
| *Nuclear* | |  |
| HCAG_02975 | Single-strand binding protein | single-stranded DNA binding, DNA replication |
| *Cytoskeletal* | |  |
| HCAG_04706 | ARP2/3 complex subunit | cytoskeleton, regulation of actin filament polymerization |
| *Anti-oxidant* | |  |
| HCAG_07098 | cytochrome c peroxidase | cytochrome-c peroxidase activity, response to oxidative stress, mitochondrial matrix, heme binding, oxidation reduction |
| HCAG_07445 | conserved hypothetical protein | cell redox homeostasis, peroxiredoxin activity, oxidation reduction |
| HCAG_09319 | cytochrome c peroxidase | cytochrome-c peroxidase activity, response to oxidative stress, mitochondrial matrix, heat shock protein binding, heme binding, oxidation reduction, nucleus, zinc ion binding |
| *Chaperone-like* | |  |
| HCAG_00783 | hsp88-like protein | ATP binding, response to stress |
| HCAG_08383 | psi protein | protein folding, unfolded protein binding, heat shock protein binding |
| *Ribosomal* | |  |
| HCAG_00055 | 60S ribosomal protein L27-A | ribosome, structural constituent of ribosome, translation |
| HCAG_02704 | 40S ribosomal protein S15 | mycelium development, structural constituent of ribosome, small ribosomal subunit, translation |
| HCAG_03415 | 60S ribosomal protein L35 | ribosome, structural constituent of ribosome, translation |
| HCAG_04185 | 60S ribosomal protein L36 | ribosome, structural constituent of ribosome, translation |
| HCAG_06613 | 40S ribosomal protein S7 | ribosome, structural constituent of ribosome, translation |
| *Miscellaneous* | |  |
| HCAG_00337 | cleavage and polyadenylation specificity factor | unknown |
| HCAG_02931 | electron transfer flavoprotein beta-subunit | oxidation reduction, oxidoreductase activity, iron ion binding, electron carrier activity, FMN binding |
| HCAG_03209 | vacuolar ATP synthase subunit E | hydrogen-exporting ATPase activity (phosphorylative mechanism), ATP synthesis coupled proton transport, proton-transporting ATPase activity (rotational mechanism), proton-transporting two-sector ATPase complex (catalytic domain) |
| HCAG_04675 | esterase D | cytoplasmic membrane-bounded vesicle, S-formylglutathione hydrolase activity, carboxylesterase activity |
| HCAG_05000 | transketolase TktA | transketolase activity, metabolic process |
| HCAG_05094 | 2-methylcitrate dehydratase | 2-methylcitrate dehydratase activity, propionate catabolic process, 2-methylcitrate cycle, 2 iron-2 sulfur cluster binding |
| HCAG_05311 | succinyl-CoA:3-ketoacid-coenzyme A transferase | ketone body catabolic process, mitochondrion, 3-oxoacid CoA-transferase activity |
| HCAG_07568 | choline sulfatase | metabolic process, choline-sulfatase activity |
| HCAG_07700 | xanthine phosphoribosyltransferase | nucleoside metabolic process, transferase activity (transferring glycosyl groups) |
| HCAG_08290 | gamma-butyrobetaine dioxygenase | oxidoreductase activity, electron carrier activity |
| HCAG_08408 | HAD-superfamily hydrolase | isomerase activity, metabolic process, hydrolase activity |
| *Biological process unclassified* | |  |
| HCAG_01198 | conserved hypothetical protein | undefined |
