# Supplementary material for: Histoplasma capsulatum Heat-Shock 60 Orchestrates the Adaptation of the Fungus to Temperature Stress
Source: PLoS One. 2011 Feb 10;6(2):e14660. doi: 10.1371/journal.pone.0014660 (PMC3037374; doi:10.1371/journal.pone.0014660)
Supplement: Table S2 — Cell wall Hsp60 interactome under different temperature stress conditions. (0.16 MB DOC) [file pone.0014660.s002.doc]

**Table S2:** Cell wall Hsp60 interactome under different temperature stress conditions.

|  |  |  |
| --- | --- | --- |
| **ID number** | **Protein name** | **Molecular Function (Gene Onthology)** |
| **30, 37, 37/40^o^C** | |  |
| *Protein metabolism and modification* | |  |
| HCAG_04485 | peptidylprolyl isomerase | protein folding, cytoplasm, peptidyl-prolyl cis-trans isomerase activity |
| HCAG_05988 | hypothetical protein similar to elongation factor 2 | translation elongation factor activity, GTPase activity, GTP binding |
| HCAG_08798 | translation elongation factor 1-alpha | cytoplasm, translation elongation factor activity, GTP binding, translational elongation, GTPase activity |
| *Carbohydrate metabolism* | |  |
| HCAG_00010 | fructose 1,6-biphosphate aldolase | glycolysis, fructose-bisphosphate aldolase activity, zinc ion binding |
| HCAG_03969 | malate dehydrogenase | binding, L-malate dehydrogenase activity, malate metabolic process, oxidation reduction, tricarboxylic acid cycle, glycolysis |
| HCAG_04910 | glyceraldehyde-3-phosphate dehydrogenase | cytoplasm, NAD or NADH binding, response to stress, oxidation reduction, glycolysis, glyceraldehyde-3-phosphate dehydrogenase (phosphorylating) activity, |
| HCAG_05266 | aconitase | aconitate hydratase activity, tricarboxylic acid cycle, 4 iron, 4 sulfur cluster binding |
| HCAG_06901 | malate dehydrogenase | binding, L-malate dehydrogenase activity, malate metabolic process, oxidation reduction, glycolysis |
| Nuclear |  |  |
| HCAG_03525 | histone H2b | nucleosome, DNA binding, nucleosome assembly, nucleus |
| *Cell wall architecture* | |  |
| HCAG_00683 | woronin body major protein | translational initiation, translation initiation factor activity, Septal pore sealing in response |
| *Chaperone-like* | |  |
| HCAG_00806 | heat shock 70 kDa protein | response to stress, oxidation reduction, ATP binding, translation, 2-alkenal reductase activity |
| HCAG_01398 | hsp70-like protein | ATP binding, response to stress |
| HCAG_04686 | ATP-dependent molecular chaperone HSC82 | protein folding, unfolded protein binding, ATP binding, response to stress |
| HCAG_08176 | heat shock protein SSC1 | protein folding, endonuclease activity, response to stress, oxidation reduction, ATP binding, unfolded protein binding, mitochondrion, 2-alkenal reductase activity |
| *Ribosomal* | |  |
| HCAG_02704 | 40S ribosomal protein S15 | mycelium development, structural constituent of ribosome, small ribosomal subunit, translation |
| HCAG_04418 | 40S ribosomal protein S24 | ribosome, structural constituent of ribosome, translation, nucleotide binding |
| *Miscellaneous* | |  |
| HCAG_02813 | ATP synthase subunit alpha | proton-transporting ATP synthase complex, catalytic core F(1), hydrogen ion transporting ATP synthase activity (rotational mechanism), mycelium development, ATP synthesis coupled proton transport, ATP binding, proton-transporting ATPase activity (rotational mechanism) |
| HCAG_04173 | 14-3-3 family protein | protein domain specific binding |
| HCAG_06944 | mitochondrial ATP synthase | proton-transporting ATP synthase complex, catalytic core F(1), hydrogen-exporting ATPase activity, phosphorylative mechanism, hydrogen ion transporting ATP synthase activity, rotational mechanism, ATP synthesis coupled proton transport, ATP binding, proton-transporting ATPase activity, rotational mechanism |
|  |  |  |
| **30, 37^o^C** |  |  |
| HCAG_05565 | cobalamin-independent methionine synthase MetH/D | methionine biosynthetic process, zinc ion binding, 5-methyltetrahydropteroyltriglutamate-homocysteine S-methyltransferase activity |
| *Cytoskeletal* | |  |
| HCAG_01781 | tubulin beta chain | microtubule-based movement, protein complex, GTP binding, protein polymerization, response to antibiotic, structural molecule activity, GTPase activity, microtubule |
| *Ribosomal* |  |  |
| HCAG_07708 | 60S ribosomal protein L13 | ribosome, structural constituent of ribosome, translation |
|  |  |  |
| **30, 40^o^C** |  |  |
| *Amino acid metabolism* | |  |
| HCAG_08890 | ketol-acid reductoisomerase | ketol-acid reductoisomerase activity, mycelium development, oxidation reduction, branched chain family amino acid biosynthetic process, isomerase activity, coenzyme binding |
| *Plasma membrane* | |  |
| HCAG_06977 | Plasma membrane ATPase | hydrogen-exporting ATPase activity, phosphorylative mechanism, integral to membrane, ATP biosynthetic process, ATP binding, magnesium ion binding, proton transport, plasma membrane |
| *Ribosomal* | |  |
| HCAG_00468 | 60S ribosomal protein L4-A | ribosome, mycelium development, structural constituent of ribosome, translation |
| HCAG_06613 | 40S ribosomal protein S7 | ribosome, structural constituent of ribosome, translation |
| HCAG_10915 | 40S ribosomal protein S8e | hydrolase activity |
| *Miscellaneous* | |  |
| HCAG_06283 | adp_ATP carrier protein | ribosome, binding, mitochondrial inner membrane, transport, integral to membrane, transporter activity |
|  |  |  |
| **37, 40^o^C** |  |  |
| *Protein metabolism and modification* | |  |
| HCAG_06295 | KH domain RNA-binding protein | growth or development of symbiont on or near host, RNA binding |
| HCAG_08833 | peptidyl-prolyl cis-trans isomerase | protein folding, cytoplasm, peptidyl-prolyl cis-trans isomerase activity, binding |
| *Carbohydrate metabolism* | |  |
| HCAG_01360 | pyruvate dehydrogenase E1 component alpha subunit | pyruvate dehydrogenase (acetyl-transferring) activity, intracellular membrane-bounded organelle, oxidation reduction, glycolysis |
| HCAG_05090 | 2-methylcitrate synthase | cellular carbohydrate metabolic process, acyltransferase activity, propionate metabolic process, methylcitrate cycle, mitochondrial matrix, 2-methylcitrate synthase activity |
| HCAG_06317 | Succinate dehydrogenase flavoprotein subunit | FAD binding, electron transport chain, tricarboxylic acid cycle, electron carrier activity, succinate dehydrogenase (ubiquinone) activity |
| *Plasma membrane* | |  |
| HCAG_03815 | ATP synthase subunit 5 | proton-transporting ATP synthase complex (catalytic core F(1)) hydrogen ion transporting ATP synthase activity (rotational mechanism), ATP synthesis coupled proton transport, proton-transporting ATPase activity (rotational mechanism) |
| *Chaperone-like* | |  |
| HCAG_05805 | heat shock 70 kDa protein C precursor | endoplasmic reticulum lumen, secretion by cell, oxidation reduction, response to unfolded protein, ATP binding, endoplasmic reticulum, 2-alkenal reductase activity |
| *Ribosomal* | |  |
| HCAG_03415 | 60S ribosomal protein L35 | ribosome, structural constituent of ribosome, translation |
| *Miscellaneous* | |  |
| HCAG_03323 | fumarate reductase flavoprotein subunit | heme binding, oxidation reduction, succinate dehydrogenase activity, electron carrier activity, fumarate reductase (NADH) activity |
| HCAG_06996 | processing/enhancing protein | oxidoreductase activity, metalloendopeptidase activity, proteolysis, |
| HCAG_08720 | mannitol-1-phosphate dehydrogenase | oxidation reduction, mannitol-1-phosphate 5-dehydrogenase activity, coenzyme binding, D-iditol 2-dehydrogenase activity |
|  |  |  |
| **30^o^C** |  |  |
| *Amino acid metabolism* | |  |
| HCAG_02120 | fumarylacetoacetase hydrolase family protein | fumarylacetoacetase activity, tyrosine catabolic process, calcium ion binding, magnesium ion binding, L-phenylalanine catabolic process |
| *Carbohydrate metabolism* | |  |
| HCAG_04934 | ATP-citrate synthase subunit 1 | cytoplasm, cellular carbohydrate metabolic process, acyltransferase activity, ATP citrate synthase activity, lipid biosynthetic process, ATP binding, magnesium ion binding, succinate-CoA ligase (ADP-forming) activity, lyase activity |
| HCAG_10958 | isocitrate lyase | peroxisome, methylisocitrate lyase activity, tricarboxylic acid cycle, glyoxysome, isocitrate lyase activity, glyoxylate cycle, peroxisome, methylisocitrate lyase activity, tricarboxylic acid cycle, isocitrate lyase activity, glyoxylate cycle |
| *Lipid, fatty acid and steroid metabolism* | |  |
| HCAG_01606 | acetate-CoA ligase | acetoin catabolic process, acetate-CoA ligase activity, AMP binding |
| HCAG_07636 | fatty acid synthase alpha subunit FasA | fatty acid biosynthetic process, holo-[acyl-carrier-protein] synthase activity, fatty-acyl-CoA synthase activity, magnesium ion binding, macromolecule biosynthetic process |
| HCAG_09197 | glycerophosphoryl diester phosphodiesterase family protein | glycerophosphodiester phosphodiesterase activity, glycerol metabolic process, lipid metabolic process |
| *Cell growth/division* | |  |
| HCAG_02006 | septin-3 | GTP binding, protein binding, cell cycle, septin complex, cell division |
| *Nuclear* | |  |
|  |  |  |
| HCAG_03885 | histone h4 | nucleosome, mycelium development, DNA binding, nucleosome assembly, nucleus |
| *Cytoskeletal* | |  |
| HCAG_08210 | actin | cytoskeleton, cytoplasm, ATP binding, protein binding |
| HCAG_08288 | tubulin subunit alpha-2 | microtubule-based movement, protein complex, GTP binding, protein polymerization, structural molecule activity, GTPase activity, microtubule |
| *Cell Wall architecture* | |  |
| HCAG_00193 | beta-glucosidase 1 | carbohydrate metabolic process, beta-glucosidase activity |
| HCAG_07031 | G-protein comlpex beta subunit CpcB | cell wall systhesis and morphogenesis, putative receptor for protein kinase C in the regulation of actin cytoskeleton organization during cell wall synthesis |
| *Anti-oxidant* | |  |
| HCAG_08064 | catalase B | heme binding, oxidation reduction, catalase activity, hydrogen peroxide catabolic process |
| HCAG_09319 | cytochrome c peroxidase | cytochrome-c peroxidase activity, response to oxidative stress, mitochondrial matrix, heat shock protein binding, heme binding, oxidation reduction, nucleus, zinc ion binding |
| *Ribosomal* | |  |
| HCAG_02430 | 40S ribosomal protein S5-A | structural constituent of ribosome, small ribosomal subunit, translation |
| HCAG_03504 | ribosomal protein L22 | ribosome, nucleobase, nucleoside, nucleotide and nucleic acid metabolic process, DNA binding, structural constituent of ribosome, ATP-dependent DNA helicase activity, ATP binding, nucleus, translation |
| HCAG_03695 | 60S ribosomal protein L10-B | ribosome, structural constituent of ribosome, translation |
| HCAG_05221 | ribosomal protein L7a | ribosome, ribosome biogenesis |
| HCAG_06425 | ribosomal protein L32 | ribosome, structural constituent of ribosome, translation |
| HCAG_07237 | ribosomal protein S4 | ribosome, mycelium development, structural constituent of ribosome, RNA binding, translation |
| HCAG_08444 | 60S ribosomal protein L5 | ribosome, 5S rRNA binding, structural constituent of ribosome, translation |
| HCAG_08667 | 40S ribosomal protein S18 | ribosome, mycelium development, structural constituent of ribosome, RNA binding, translation |
| *Miscellaneous* | |  |
| HCAG_04527 | 14-3-3 family protein ArtA | protein domain specific binding |
| HCAG_04999 | spermidine synthase | spermidine synthase activity, transferase activity |
| HCAG_05099 | nascent polypeptide-associated complex subunit alpha | cytoplasm, nucleus, regulation of transcription, DNA-dependent, protein transport |
| HCAG_08367 | aldehyde dehydrogenase | oxidation reduction, aldehyde dehydrogenase (NAD) activity |
|  |  |  |
| **37^o^C** |  |  |
| *Protein metabolism and modification* | |  |
| HCAG_09244 | nuclear and cytoplasmic polyadenylated RNA-binding protein pub1 | nucleic acid binding, nucleotide binding |
| *Carbohydrate metabolism* | |  |
| HCAG_08493 | fumarate hydratase class II | fumarate hydratase activity, tricarboxylic acid cycle enzyme complex, fumarate metabolic process |
| *Cell growth/division* | |  |
| HCAG_02452 | cell division cycle protein | nucleoside-triphosphatase activity, cell cycle, protein transport, ATP binding, response to stress, cell division |
| *Ribosomal* |  |  |
| HCAG_06308 | 40S ribosomal protein S12 | ribosome, structural constituent of ribosome, translation |
| HCAG_08515 | 60S ribosomal protein L2 | ribosome, structural constituent of ribosome, translation |
| *Miscellaneous* | |  |
| HCAG_03209 | vacuolar ATP synthase subunit E | hydrogen-exporting ATPase activity (phosphorylative mechanism), ATP synthesis coupled proton transport, proton-transporting ATPase activity (rotational mechanism), proton-transporting two-sector ATPase complex (catalytic domain) |
| HCAG_04799 | ATP synthase gamma chain | proton-transporting ATP synthase complex (catalytic core F(1)), hydrogen ion transporting ATP synthase activity (rotational mechanism), ATP synthesis coupled, proton-transporting ATPase activity (rotational mechanism)growth or development of symbiont on or near hostproton transport |
|  |  |  |
| **40^o^C** |  |  |
| *Protein metabolism and modification* | |  |
| HCAG_01784 | elongation factor 1-beta | translation elongation factor activity, eukaryotic translation elongation factor 1 complex, translational elongation |
| HCAG_03444 | elongation factor Tu | translation elongation factor activity, GTP binding, intracellular, translational elongation, GTPase activity |
| HCAG_06026 | polyadenylate-binding protein | cytoplasm, RNA binding, mRNA transport, mRNA processing, regulation of translation, nucleus, nucleotide binding |
| *Carbohydrate metabolism* | |  |
| HCAG_03972 | dihydrolipoamide acetyltransferase component | pyruvate dehydrogenase complex, pyruvate metabolic process, dihydrolipoyllysine-residue acetyltransferase activity, protein binding, intracellular protein transport, lipoic acid binding, |
| *Nuclear* | |  |
| HCAG_02914 | glycine-rich protein | nucleic acid binding, mycelium development, nucleotide binding |
| HCAG_04410 | RNA-binding protein | nucleic acid binding, nucleotide binding |
| *Proteasome component* | |  |
| HCAG_00039 | 26S protease regulatory subunit | peptidase activity, protein complex, protein catabolic process, ATP binding, nucleoside-triphosphatase activity, nucleus, cytosol, growth or development of symbiont on or near host |
| *Chaperone-like* | |  |
| HCAG_04111 | heat shock protein 30 | response to stress |
| HCAG_04471 | heat shock protein STI1 | response to stress, binding |
| *Ribosomal* | |  |
| HCAG_01947 | ribosomal protein S23 | structural constituent of ribosome, small ribosomal subunit, translation |
| HCAG_03167 | 60S ribosomal protein L24 | ribosome, structural constituent of ribosome, translation |
| HCAG_04519 | 60S ribosomal protein L14p/L23e | ribosome, peptidase activity, structural constituent of ribosome, translation |
| HCAG_06361 | Ribosomal protein S28e | transferase activity (transferring glycosyl groups), structural constituent of ribosome, cytosolic small ribosomal subunit, translation |
| *Miscellaneous* | |  |
| HCAG_00437 | cytochrome c oxidase chain VI | cytochrome-c oxidase activity |
| HCAG_05306 | RNP domain-containing protein | nucleic acid binding, nucleotide binding |
| HCAG_06929 | NADH-ubiquinone oxidoreductase | electron carrier activity, NADH dehydrogenase (ubiquinone) activity, iron ion binding, 2 iron, 2 sulfur cluster binding, 4 iron, 4 sulfur cluster binding, ATP synthesis coupled electron transport, membrane |
| *Biological process unclassified* | |  |
| HCAG_06889 | Transcriptional repressor TUP1 | undefined |
