# Supplementary figures and images for: Histoplasma capsulatum Heat-Shock 60 Orchestrates the Adaptation of the Fungus to Temperature Stress
Source: PLoS One. 2011 Feb 10;6(2):e14660. doi: 10.1371/journal.pone.0014660 (PMC3037374; doi:10.1371/journal.pone.0014660)

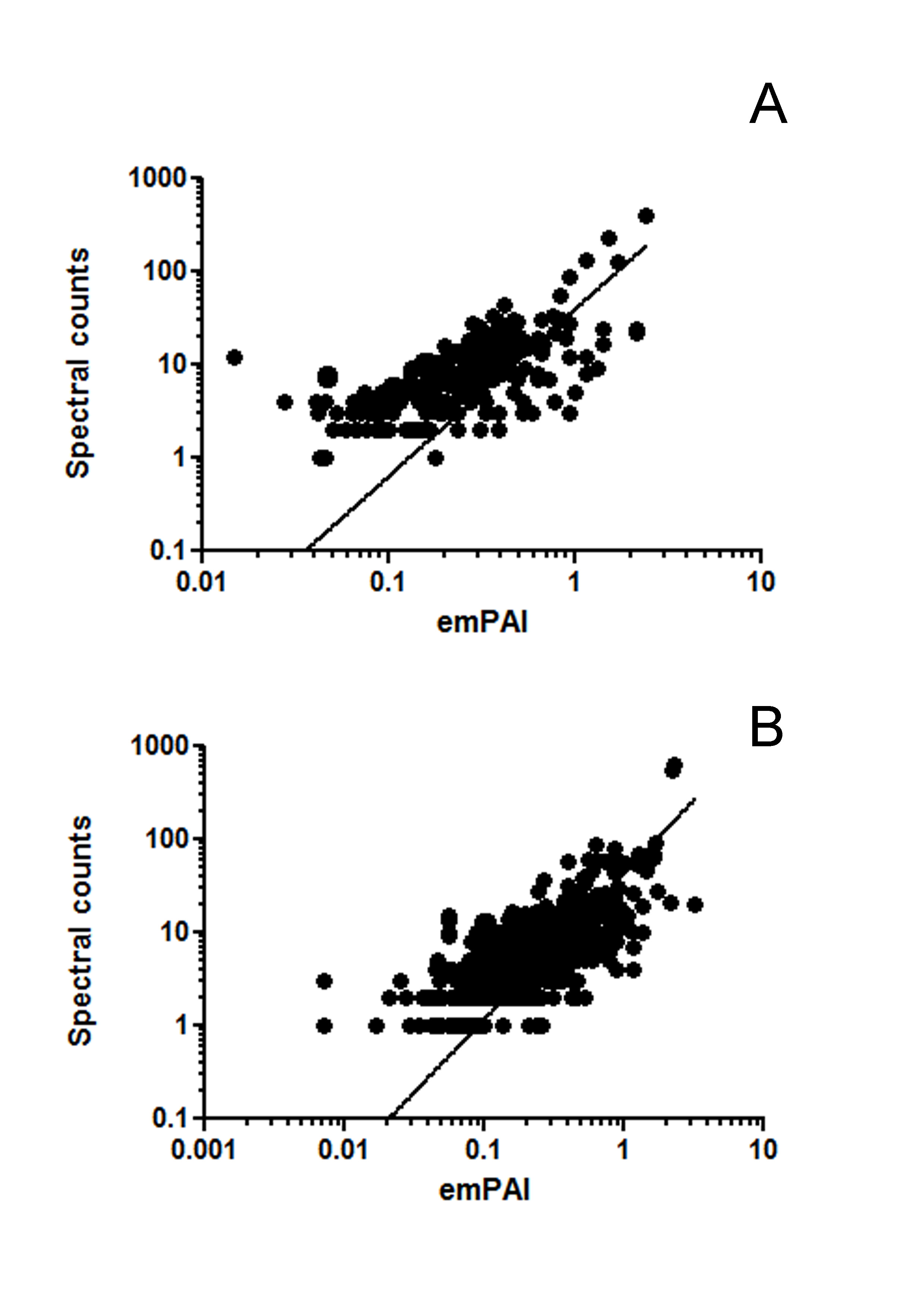

Supplement: Figure S1 — Correlations of the two parameters used to quantitatively evaluate the mass spectrometry analyses, emPAI and spectral counts. Correlation of emPAI and spectral counts for all of the proteins identified in the (A) cytoplasmic fraction and (B) cell wall fraction. (0.67 MB TIF) [file pone.0014660.s003.tif]

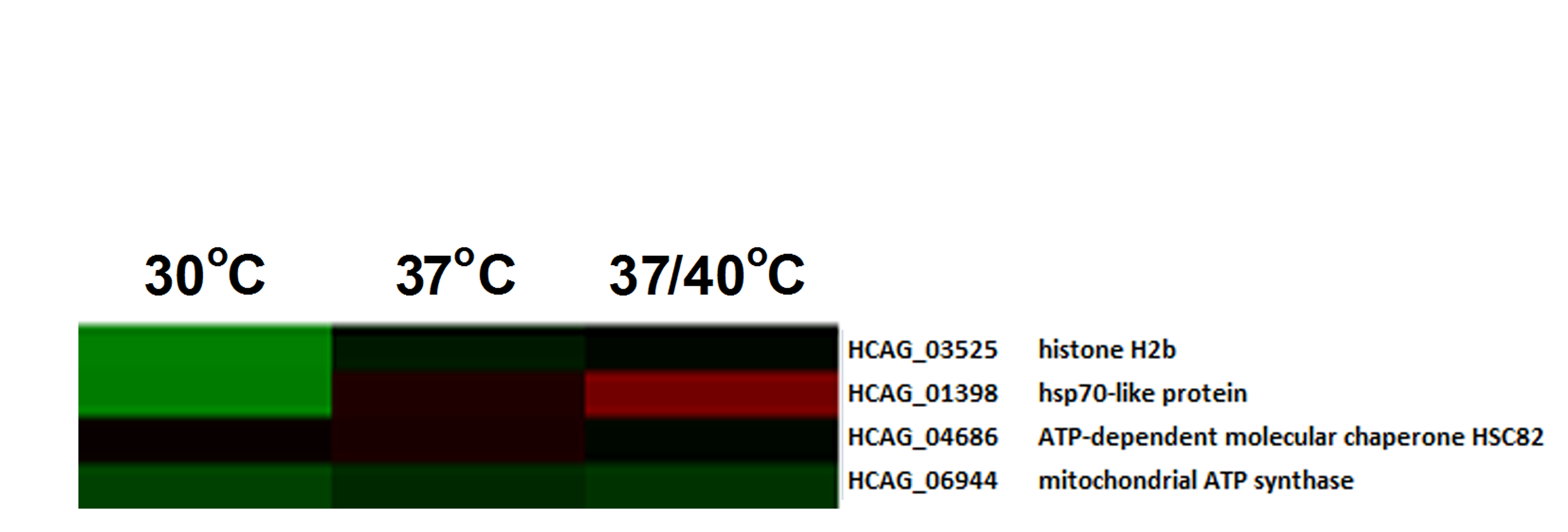

Supplement: Figure S2 — Graphic representation of the levels of interaction of Hsp60 with distinct interaction partners in the cytoplasm at different temperature conditions. Results illustrate that differences were observed for the majority of common proteins identified, as shown in Table 3. (0.35 MB TIF) [file pone.0014660.s004.tif]

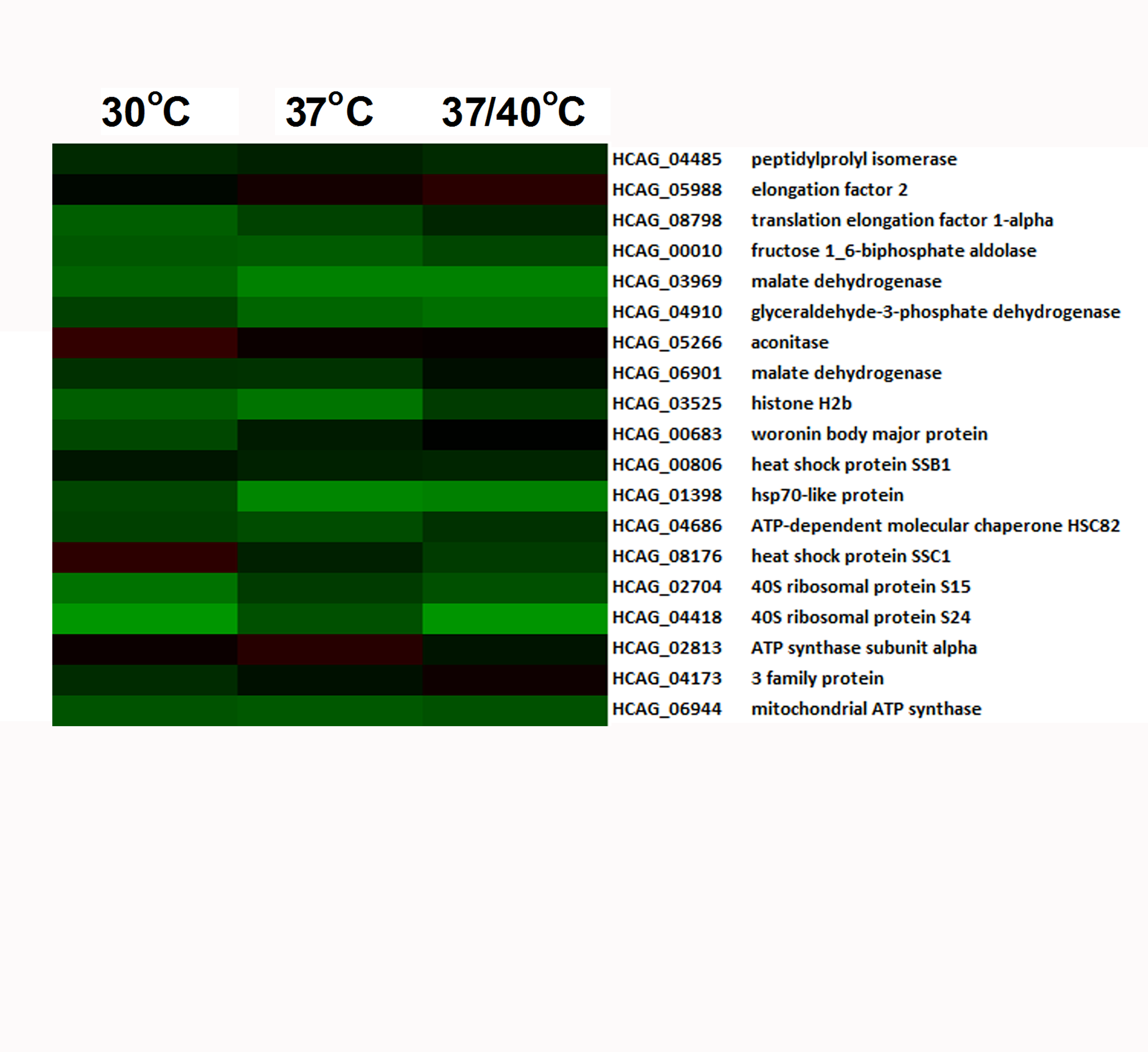

Supplement: Figure S3 — Graphic representation of levels of interaction of the Hsp60 with distinct interaction partners in the cell wall at different temperature conditions. As in Table 3, the results illustrate that there were no difference in terms of percentage of interactions for the majority of common proteins identified, suggesting that in most cases constitutive interactions occur at similar levels, independent of temperature. (1.35 MB TIF) [file pone.0014660.s005.tif]
